# Two-dimensional van der Waals heterostructures of BAs/MoSeTe and BAs/MoTeSe for photocatalytic and photovoltaic applications

**DOI:** 10.1039/d6cp03340g

**Published:** 2026-10-21

**Authors:** Hanifan BiBi, Azeem Ghulam Nabi, Aman-ur-Rehman, Devis Di Tommaso

**Affiliations:** a Department of Physics, Allama Iqbal Open University H-8 Islamabad 44000 Pakistan; b Department of Physics, Hafiz Hayat Campus, University of Gujrat Gujrat 50700 Pakistan azeem.nabi@uog.edu.pk imagnabi@gmail.com; c Department of Nuclear Engineering, Pakistan Institute of Engineering & Applied Sciences Nilore Islamabad 45650 Pakistan; d Center for Mathematical Sciences, Pakistan Institute of Engineering & Applied Sciences Nilore Islamabad 45650 Pakistan; e Department of Chemistry, School of Physical and Chemical Sciences, Queen Mary University of London Mile End Road London E1 4NS UK d.ditommaso@qmul.ac.uk

## Abstract

Janus transition-metal dichalcogenide monolayers offer intrinsic out-of-plane polarisation fields that, when combined with a suitable partner layer, can enhance charge separation in van der Waals heterostructures. Here we use density functional theory to investigate the structural, electronic, optical, and photocatalytic properties of BAs/Janus-MoSeTe (H-I) and BAs/Janus-MoTeSe (H-II) heterostructures, including the effects of Se and Te chalcogen vacancies. Phonon spectra and negative formation energies confirm the dynamic and thermodynamic stability of both systems. The pristine heterostructures adopt type-II band alignments with direct interlayer band gaps of 1.24 eV (H-I) and 0.91 eV (H-II); introducing vacancies converts these to indirect gaps and narrows the interlayer band gap. Within the type-II charge-transfer pathway imposed by the interfacial field, the band edges do not straddle the water redox potentials, and the calculated hydrogen adsorption free energies (Δ*G*_H*_ ≈ 1 eV) further indicate weak hydrogen binding on the pristine surfaces, so that further surface engineering is needed to achieve competitive catalytic kinetics. H-II exhibits electron mobilities of up to ∼10^4^ cm^2^ V^−1^ s^−1^ within deformation-potential theory, and Se vacancies produce larger band offsets than Te vacancies, consistent with more effective spatial separation of photoexcited carriers. Optical absorption spans the visible and ultraviolet regions, with heterostructure formation broadening the spectral response relative to the constituent monolayers. The estimated power conversion efficiencies of approximately 25%, calculated within a Shockley–Queisser-type framework, place these systems among the most promising 2D heterostructures for photovoltaic energy conversion.

## Abstract 2

Contacting BAs through the Se or Te face of Janus MoSeTe switches the heterostructure from type-I to type-II alignment, giving near-optimal photovoltaic band gaps, while band edges alone are shown to overstate photocatalytic activity.

## Graphical abstract

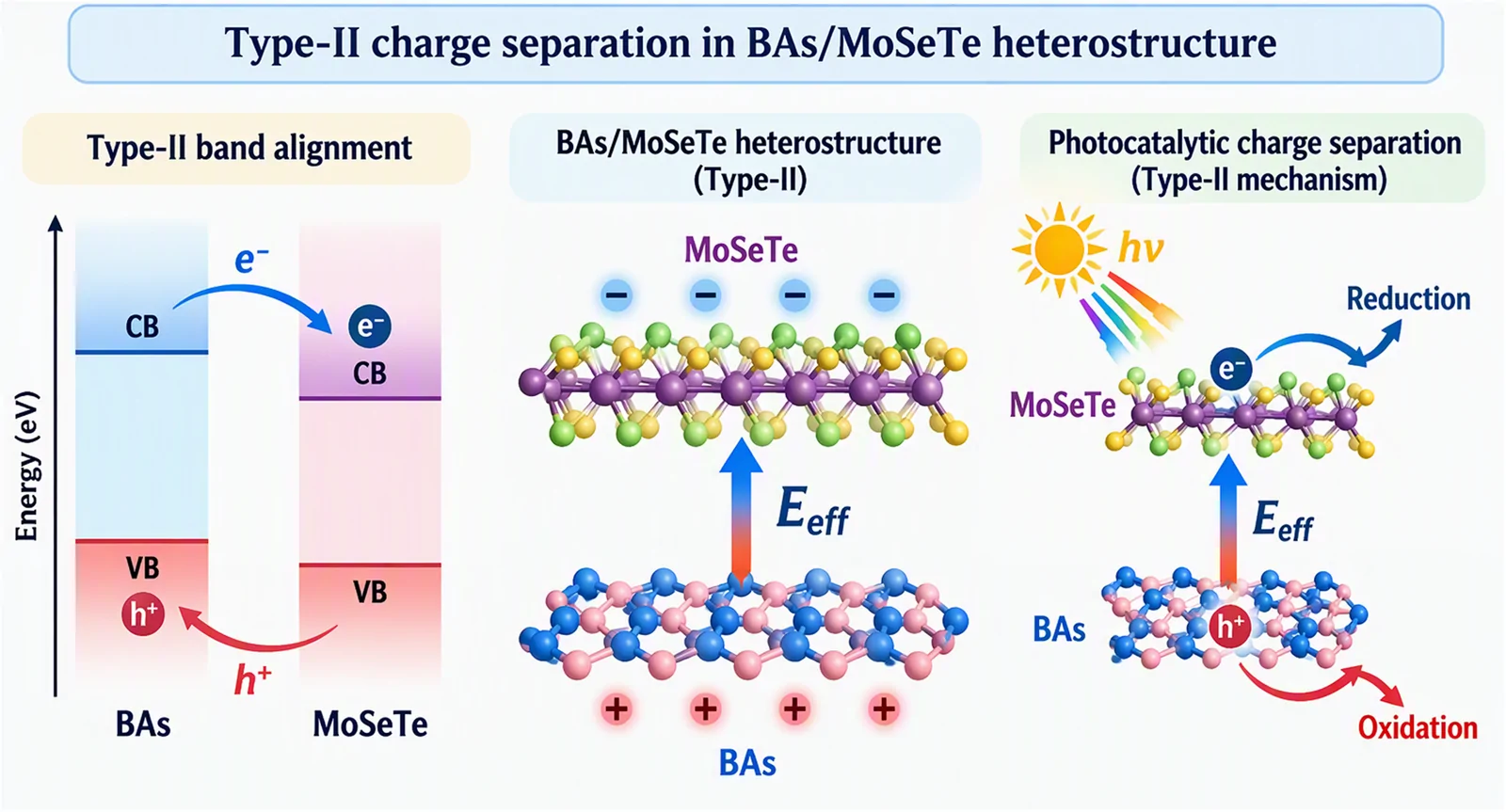

## Introduction

The inadequate availability of fossil fuels, along with their harmful environmental effects and role in climate change, has intensified environmental pollution and energy crises, presenting significant challenges for humanity.^1^ It is crucial to identify a sustainable energy source to replace these finite fossil fuels. Hydrogen produced through photocatalytic water splitting is considered a sustainable, clean, and renewable energy source for the future.^2–4^ However, selecting an appropriate photocatalyst is essential for efficient hydrogen production from water splitting. The primary obstacles for a suitable photocatalyst are a large band gap and improper alignment of the band edge positions.^5–7^

Graphene, a two-dimensional (2D) material, has been widely researched for its potential in electronic and optoelectronic devices, despite its lack of a tuneable band gap.^8^ As a result, recent years have seen extensive exploration of new types of 2D materials, such as phosphorene,^9^ transition metal dichalcogenides (TMDs),^10^ and MXenes.^11^ Compared to their bulk counterparts, TMDs offer improved charge transfer and separation capabilities due to their large specific surface area, making them promising for applications in optoelectronics, transistors, and sensors.^10^ Among TMDs, MoX_2_ (X = S, Se, Te) monolayers have been the focus of significant theoretical and experimental studies.^12^

Recent advancements in the field of 2D transition metal dichalcogenides (TMDs) have led to the synthesis of Janus MXY (M = W, Mo; X/Y = Se, S, Te) *via* sulfurisation or selenisation using the CVD technique.^13^ The MoSSe monolayer is produced by selectively selenizing the top atomic layer of a single-layer MoS_2_ structure, unlike its mirror-symmetric counterparts MoS_2_ and MoSe_2_. In Janus materials, the difference in electronegativity between sulfur and selenium atoms induces an intrinsic out-of-plane polarisation, enabling coupling with the natural interlayer built-in polarization field. These materials have garnered significant interest due to their unique properties and potential applications, offering an additional degree of flexibility for tuning their physicochemical properties. Moreover, Janus-MoSeTe and Janus-MoTeSe monolayers are direct band gap semiconductors with moderate band gaps.^14^ Unfortunately, not all 2D materials are suitable for water splitting, as many have large band gaps and band edge alignments that are far from the redox potentials.

van der Waals heterostructures (vdWHs) offer a route to engineer band-edge positions suitable for water splitting while retaining the desirable properties of the constituent monolayers.^14–20^ Among the Group III–V arsenide semiconductors,^21–27^ the hexagonal boron arsenide (BAs) monolayer has attracted particular attention as a vdWH building block for photocatalytic and photovoltaic applications.^28^ BAs combines a direct band gap of 1.38 eV, high electron and hole mobilities exceeding 5000 cm^2^ V^−1^ s^−1^, strong in-plane mechanical stiffness, and an ultrahigh thermal conductivity of ∼2000 W m^−1^ K^−1^ at room temperature,^29^ properties that are complementary to those of TMD monolayers with narrower band gaps and high optical absorption. Accordingly, BAs has been paired with MoTe_2_,^6^ MoSe_2_,^30^ SnC,^31^ GaN^32^ and MoSi_2_N_4_^33^ to form vdWHs, with Cao *et al.* reporting a direct Z-scheme mechanism for MoTe_2_/BAs^6^ and Guan *et al.* predicting a power conversion efficiency of 3% for BAs/WSe_2_.^28,34^ However, none of these studies have examined BAs paired with a Janus TMD monolayer, whose broken mirror symmetry introduces an intrinsic out-of-plane dipole that may modify interfacial charge transfer relative to the symmetric analogues.

While BAs-based van der Waals heterostructures have shown photovoltaic and photocatalytic potential, including MoTe_2_/BAs^6^ and BAs/WX_2_ (X = S, Se),^35^ the effect of replacing the symmetric MoTe_2_ monolayer with Janus MoSeTe or MoTeSe, whose broken mirror symmetry introduces an intrinsic out-of-plane electric field, has not been explored.

This study uses density functional theory to investigate the structural, electronic, optical, and photocatalytic properties of BAs/Janus-MoSeTe (H-I) and BAs/Janus-MoTeSe (H-II) heterostructures, employing hybrid (HSE06) and quasiparticle (GW) corrections to obtain reliable band gaps and optical spectra. We examine how the orientation of the Janus dipole relative to the interface, *i.e.* contact through the Se (H-I) or Te (H-II) face, controls the interfacial charge transfer and the resulting band alignment, which is type-II for H-II and type-I for H-I, where both band edges reside on BAs. We further assess how Se or Te chalcogen vacancies modify the band gap, band offsets and carrier effective masses, with Se vacancies producing larger changes than Te vacancies. Finally, we evaluate the carrier mobilities, optical absorption and theoretical power conversion efficiencies of the pristine heterostructures, which identify BAs/Janus-MoSeTe systems as promising candidates for photovoltaic applications.

## Methods

All DFT computations were conducted using the Vienna *ab initio* simulation package (VASP, version 6.4) and the projector augmented wave (PAW) method. To accurately describe the van der Waals interactions between BAs and Janus-MoSeTe or Janus-MoTeSe monolayers, geometry optimisation and *ab initio* molecular dynamics (AIMD) simulations were performed using the Perdew–Burke–Ernzerhof (PBE) generalized gradient approximation (GGA) along with Grimme's D3 functional (PBE-D3).^36^

To investigate the effect of chalcogen vacancies, a single Se or Te atom was removed from the supercell of the most stable H-I (stacking-D) and H-II (stacking-F) configurations, corresponding to one vacancy per 16 formula units. The vacancy structures were fully re-optimised at the PBE-D3 level using the same convergence criteria and *k*-mesh as the pristine systems. Spin-polarised calculations were performed to check for vacancy-induced magnetic moments.

The optical properties of the mentioned heterostructures and monolayers were also calculated using the hybrid HSE06 (Heyd–Scuseria–Ernzerhof) functional and GW approximation to overcome the issue of PBE, which underestimates semiconductor band gaps. Convergence criteria for energy and force were set at 10^−6^ eV and 0.01 eV Å^−1^, respectively. A *k*-point (Monkhorst–Pack) grid of (15 × 15 × 1 for PBE and 12 × 12 × 1 for HSE06) and a cutoff energy of 520 eV were used to ensure computational accuracy. A vacuum space of 30 Å was maintained to minimize interactions between adjacent layers. AIMD simulations were performed using a Nosé–Hoover thermostat with a (3 × 3 × 1) supercell at the Γ point for 5 ps using a 2 fs timestep in an NVT ensemble at 300 K. Phonon dispersion curves were calculated using a 4 × 4 × 1 supercell within the framework of the frozen-phonon approximation. Carrier mobilities were evaluated within the deformation potential framework.^35^ Spin–orbit coupling (SOC) was not included. In Mo-based dichalcogenides, SOC mainly splits the valence band at K (by ≈0.2 eV), while the conduction-band splitting is only a few tens of meV;^37^ given that the CBM of both heterostructures lies on the BAs layer, inclusion of SOC is expected to lower the interlayer gaps by ≈0.1 eV without altering the band-alignment type or the qualitative trends discussed below. Uniaxial strains of ±0.5%, ±1.0%, and ±1.5% were applied along the armchair and zigzag directions, and the resulting band-edge shifts and total energy changes were used to extract deformation potential constants (*E*_1*i*_) and in-plane elastic moduli (C_2_D), as detailed in Fig. S1 and S2 of the SI using PBE-D3 for the strained-cell calculations. Band-edge energies were referenced to the vacuum level of each strained cell. Strains along the armchair and zigzag directions were applied to the orthorhombic (rectangular) cell, with the strain defined relative to the equilibrium length of the corresponding cell vector, and *C*_2D_ was normalised to the equilibrium area *S*_0_ of the cell used. DP theory considers only scattering by longitudinal acoustic phonons and neglects optical-phonon, polar and intervalley scattering; the resulting mobilities are therefore upper bounds, appropriate for comparing systems treated at the same level of theory. Free energies of the HER and OER intermediates were calculated within the computational hydrogen electrode model, Δ*G* = Δ*E* + Δ*E*_ZPE_ − *T*Δ*S*, with adsorption on the [Janus/BAs] surface of a [4 × 4] supercell.

## Results and discussion

### Structural properties

The Janus MoSeTe monolayer has lattice constants *a* = *b* = 3.43 Å, with bond lengths of 2.55 Å and 2.71 Å between Mo–Se and Mo–Te atoms, respectively. The Pauling electronegativities are 2.16 (Mo), 2.55 (Se), and 2.10 (Te). The difference in electronegativity between Mo and Se (0.39) suggests a moderate polar covalent bond, while the difference between Mo and Te (0.06) indicates a more metallic or less polar bond. The calculated lattice constant of 3.43 Å and Mo–Se and Mo–Te bond lengths of 2.55 and 2.71 Å for the Janus MoSeTe monolayer agree with the values reported by Cao *et al.*^6^ (3.43 Å, 2.54 Å, and 2.72 Å) and Zhou *et al.*^38–40^ The BAs with Janus-MoSeTe and BAs with Janus-MoTeSe monolayers are used to construct 12 vdW heterostructure configurations. These configurations are divided into two categories: (i) BAs/Janus MoSeTe vdWHs, where the BAs monolayer is adjacent to the Se atom of the Janus-MoSeTe monolayer; (ii) BAs/Janus MoTeSe vdWHs, where the BAs layer is adjacent to the Te atoms of the Janus MoTeSe monolayer. For each of these two categories, six different stacking configurations are possible, as illustrated in Fig. 1. These two categories correspond to the two inequivalent ways of placing BAs on the Janus monolayer, *i.e.* on its Se-terminated or Te-terminated face. Since a rigid inversion of the whole bilayer along the out-of-plane direction leaves the structure unchanged, they exhaust the distinct top/bottom arrangements and differ in the orientation of the intrinsic Janus dipole relative to the interface.

**Fig. 1:**
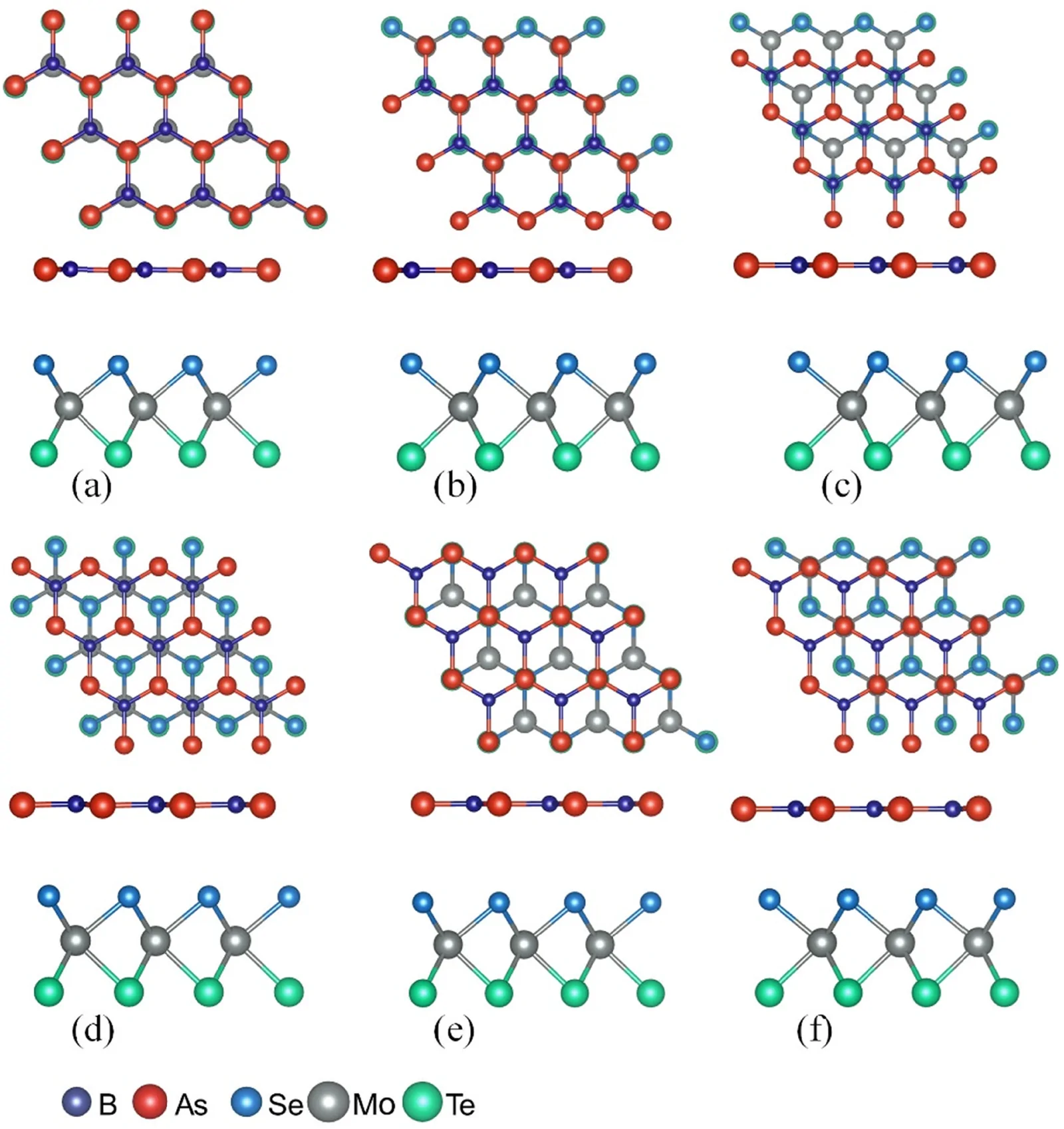
Top and side views of the six stacking configurations for the BAs/Janus-MoSeTe (H-I) van der Waals heterostructure: (a) B and As atoms atop Mo and Se atoms; (b) B and As atoms atop Se and Mo atoms; (c) As at the hollow site, B atop Se; (d) As at the hollow site, B atop Mo; (e) As atop Se, B at the hexagonal centre; (f) As atop Mo, B at the hexagonal centre. The BAs/Janus-MoTeSe (H-II) configurations follow the same six registries with BAs placed on the opposite (Te-terminated) face of the Janus monolayer.

The computed lattice parameters and interlayer distances of the optimised BAs/MoSeTe and BAs/MoTeSe vdWHs are listed in Table 1. Moreover, their formation energies (Δ*E*) were evaluated according to:

1 Δ*E* = *E*_heter_ − *E*_layer1_ − *E*_layer2_

where *E*_heter_, *E*_layer1_, and *E*_layer2_ are the total energies of the heterostructure, BAs, and Janus MoSeTe monolayer, respectively. The two most stable heterostructures according to the values of formation energy are BAs/Janus MoSeTe (stacking-D) in Fig. 1(d) and BAs/Janus MoTeSe (stacking-F) vdWHs in Fig. 1(f). BAs/Janus MoSeTe (stacking-D) is more stable than the next lowest-energy configuration (stacking-F) by only 3 meV, while BAs/Janus MoTeSe (stacking-F) is favoured over stacking-D by 6 meV. Both margins are below *k*_B_*T* at 300 K, suggesting that the two lowest-energy registries may coexist under ambient conditions in both heterostructures. For H-I, stacking-D and stacking-F have almost identical interlayer distances and HSE06 band gaps (1.24 and 1.25 eV; Table 1); for H-II, stacking-D has a smaller band gap (0.70 eV) than stacking-F (0.92 eV). In the following, the lowest-energy configurations, H-I (stacking-D) and H-II (stacking-F), are used as representative models. The following structures were considered to investigate the vibrational, electronic, photocatalytic (water splitting), and optical properties: (i) BAs/Janus MoSeTe (H-I); (ii) BAs/Janus MoTeSe (H-II) and (iii) H-I and H-II with Te and Se vacancies. The absence of imaginary frequencies in the phonon spectra (Fig. 2) confirms the structural stability of BAs, MoSeTe, and the H-I and H-II heterostructures. However, AIMD simulations, without dispersion correction, indicate thermodynamic instability in these heterostructures due to monolayer separation (BAs and MoTeSe) or increased interlayer spacing (BAs and MoSeTe). The lattice parameters are consistent with previous work: 3.32 Å for monolayer BAs, 3.43 Å for MoSeTe, and 3.39 Å and 3.38 Å for the H-I and H-II heterostructures, respectively.^28^ No experimental data are yet available for these heterostructures; however, the D3 correction reproduces experimental interlayer spacings of layered materials to within a few percent,^40^ and the PBE-D3 interlayer distances obtained here (3.37 and 3.54 Å) are comparable to those of related BAs-based vdWHs.^28^

**Table 1:** Structure and energy parameters of the isolated hexagonal BAs and MoSeTe monolayers and their vdWs heterostructures: lattice parameter *a* (Å); band gap (*E*_g_), formation energy Δ*E* (eV); distance *d* (Å) between BAs and Janus-MoSeTe or Janus-MoTeSe monolayers in the twelve heterostructure configurations and with the Se or Te vacancy defects. Geometry optimization conducted at the PBE-D3 level of theory

System	*A*	*d*		*E*_g_		Δ*E*
Method		PBE	PBE-D3	HSE	PBE	
BAs	3.324	—	—	1.380	0.753	—
MoSeTe	3.436	—	—	1.778	1.35	—

BAs/MoSeTe						
Stacking-A	3.391	4.196	3.682	1.267	0.706	−0.191
Stacking-B	3.393	4.182	3.495	1.419	0.774	−0.215
Stacking-C	3.396	4.187	3.630	1.332	0.779	−0.199
Stacking-D	3.395	4.176	3.365	1.239	0.762	−0.241
Stacking-E	3.393	4.185	3.834	1.279	0.698	−0.180
Stacking-F	3.393	4.137	3.363	1.253	0.664	−0.238

BAs/MoTeSe						
Stacking-A	3.390	4.496	3.916	0.876	0.501	−0.185
Stacking-B	3.392	4.334	3.738	0.908	0.509	−0.212
Stacking-C	3.389	4.477	3.842	0.826	0.411	−0.192
Stacking-D	3.393	4.078	3.611	0.695	0.318	−0.243
Stacking-E	3.390	4.483	4.040	0.929	0.561	−0.177
Stacking-F	3.391	3.932	3.540	0.915	0.582	−0.249

BAs/MoSeTe (Se-Vac)	3.394	4.176	3.390	0.952	0.490	—
BAs/MoSeTe (Te-Vac)	3.394	4.176	3.391	1.104	0.612	—
BAs/MoTeSe (Se-Vac)	3.394	3.932	3.627	0.463	0.235	—
BAs/MoTeSe (Te-Vac)	3.394	3.932	2.951	0.880	0.575	—

**Fig. 2:**
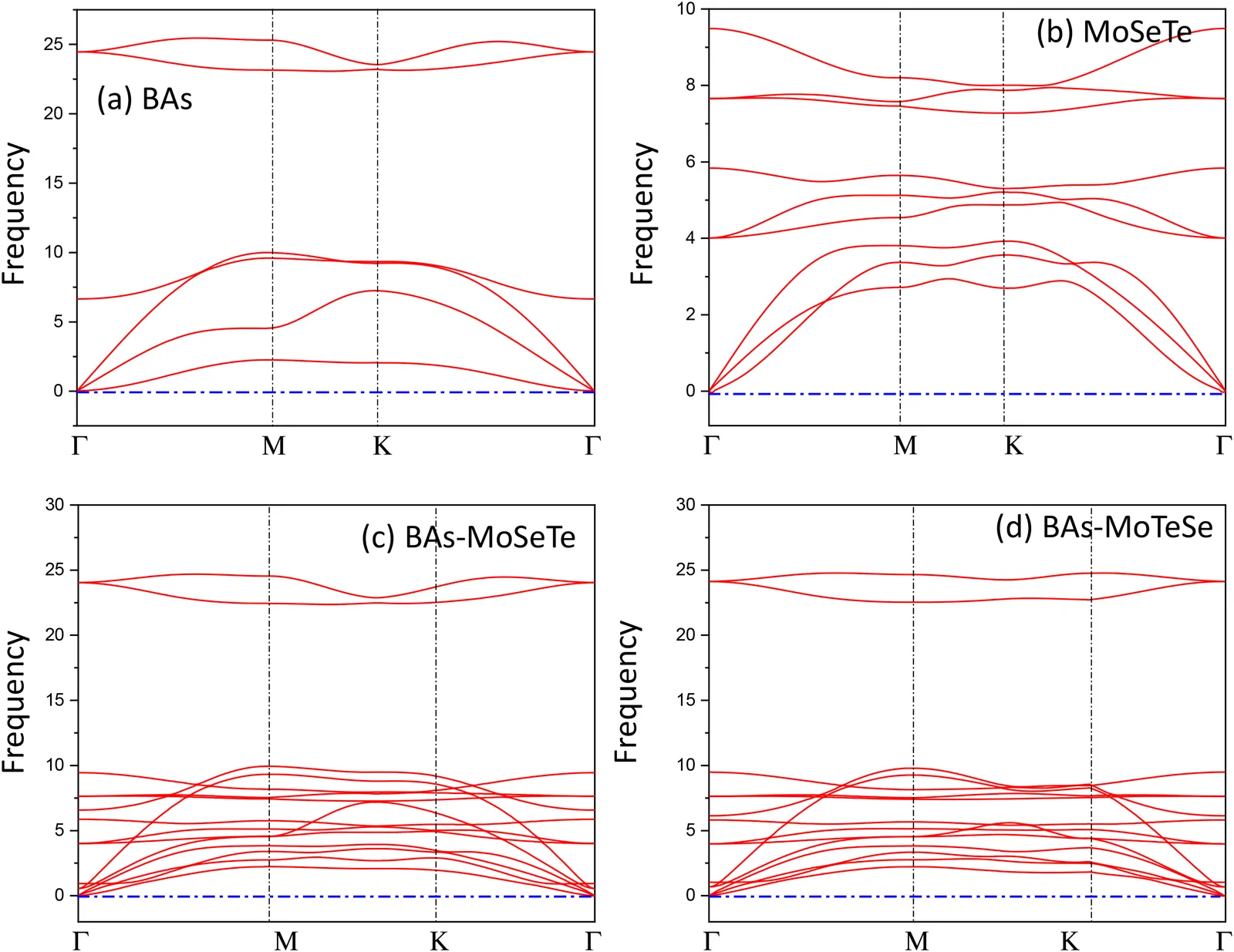
Phonon dispersion curves of (a) BAs, (b) MoSeTe, (c) H-I (BAs/MoSeTe), and (d) H-II (BAs/MoTeSe) calculated using the frozen-phonon method with a 4 × 4 × 1 supercell. The absence of imaginary frequencies confirms dynamic stability.

When stacking two single-crystalline materials, a significant difference in their lattice structures (lattice mismatch) can lead to defects at the interface. A smaller lattice mismatch is generally desirable to minimize these defects. In the case of BAs and Janus-MoSeTe, a lattice mismatch of 3.3% is reported.^41^ This level of mismatch is considered to be within a limit that should help prevent the formation of surface defects.^42^ On the other hand, inclusion of the D3 dispersion correction reduces the optimised interlayer distances by 0.4–0.8 Å relative to bare PBE (Table 1), consistent with the expected strengthening of vdW binding.^43^ At the PBE-D3 level, the most stable configurations are H-I (stacking-D, *d* = 3.365 Å) and H-II (stacking-F, *d* = 3.540 Å), which exhibit negative formation energies (−0.241 and −0.249 eV, respectively), indicating thermodynamic stability at 0 K.

### Electronic properties

The electronic band structure provides insights into the electronic structure of heterostructures. To this end, the electronic properties of BAs and Janus MoSeTe monolayers are first examined, followed by an investigation of the BAs/Janus MoSeTe or BAs/Janus MoTeSe heterostructures. Fig. 3 illustrates the band structures of BAs and Janus MoSeTe, calculated using both the PBE and HSE06 functionals. The HSE06 band gaps are 1.38 eV (BAs) and 1.78 eV (MoSeTe), while PBE yields 0.75 eV and 1.35 eV, respectively, consistent with the known underestimation of semiconductor band gaps by semi-local functionals,^44^.^7^ Additionally, both Janus MoSeTe and BAs are direct band gap semiconductors, as the conduction band minima (CBM) and valence band maxima (VBM) are located at the same high-symmetry K point in the Brillouin zone. The direct band gap semiconductor nature of these monolayers is maintained after forming heterostructures.

**Fig. 3:**
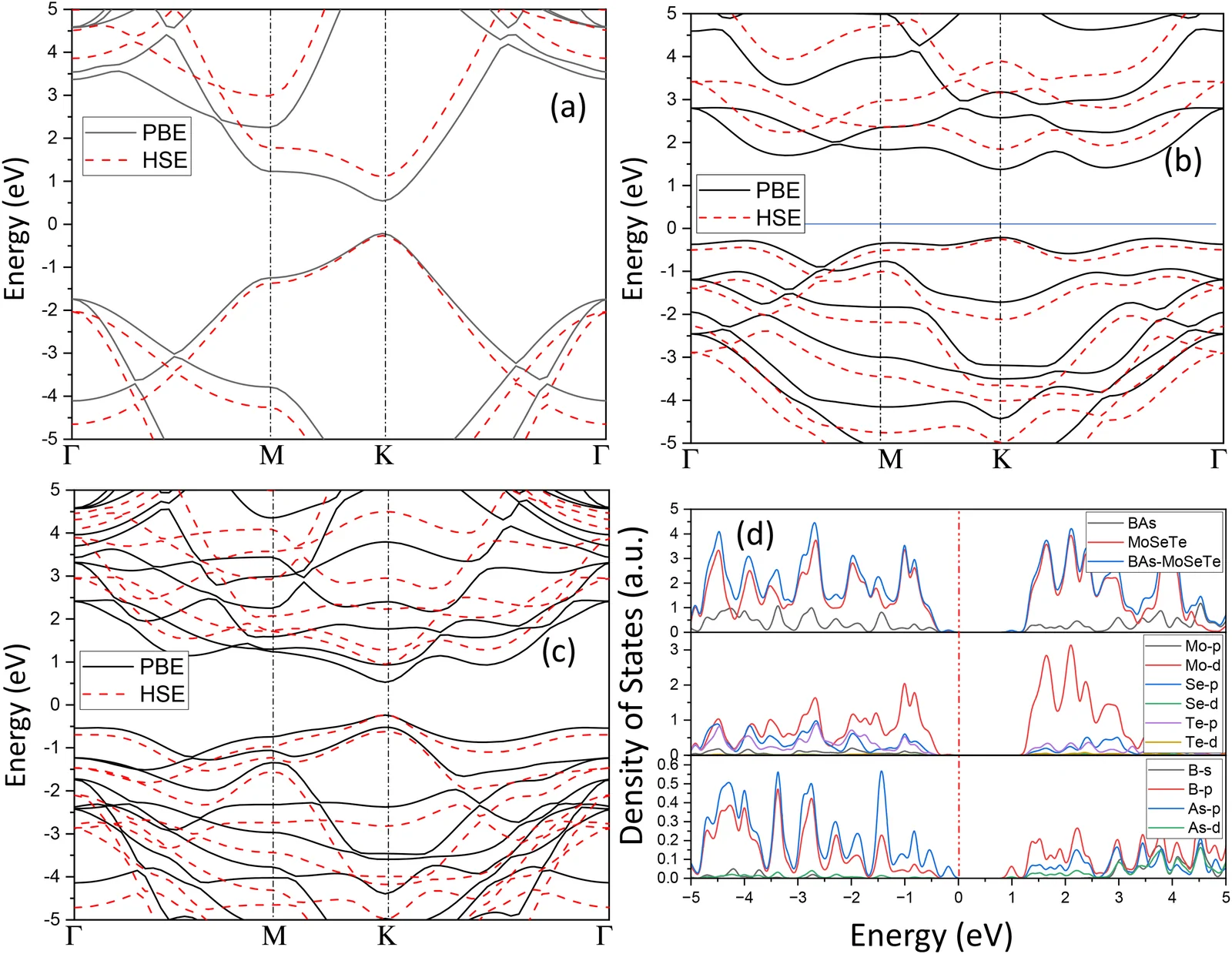
Band structures of (a) the BAs monolayer, (b) the Janus MoSeTe monolayer, and (c) the H-I heterostructure, calculated using the PBE (black solid lines) and HSE06 (red dashed lines) functionals. (d) Density of states of the H-I heterostructure at the HSE06 level: total DOS with BAs and MoSeTe contributions (top), and partial DOS projected onto the Mo, Se and Te (middle) and B and As (bottom) atoms. Differences in the DOS-free region between panels reflect the layer-resolved band edges; the energy zero is the Fermi level.

To explore the electronic structure of the heterostructures in more detail, the total density of states (TDOS) and partial density of states (PDOS) of H-I are shown in Fig. 3d. In the Janus MoSeTe layer, the valence-band edge is dominated by Mo-4d states with Se-4p and Te-5p contributions, while the conduction-band edge consists primarily of Mo-4d states.^39^ In the BAs layer, the valence-band edge is formed by As-4p states and the conduction-band edge by B-2p states, in agreement with previous studies.^29^ Because each projection contains only the states of one layer, the DOS-free region differs between the panels of Fig. 3d, and the relative positions of these layer-resolved band edges define the band alignment. For H-I, the conduction-band edge of BAs lies below that of MoSeTe, so the CBM is localised on BAs [A: whereas the valence-band edges of the two layers lie within ≈0.2 eV of each other, so that holes are only weakly localised on the Janus layer. H-I is therefore best described as a quasi-type-II heterostructure.] [B: and the valence-band edge of BAs also lies above that of MoSeTe, so that both band edges reside on BAs. H-I therefore exhibits a type-I (straddling) alignment.] This is confirmed by the layer-projected band structures in Fig. 4: in H-I the top valence band at K has predominantly BAs character with a substantial MoSeTe contribution, whereas in H-II the VBM is clearly localised on the Janus MoTeSe layer (blue) and the CBM on BAs (red), giving a type-II alignment (Δ*E*_c_ = 0.87 eV, Δ*E*_V_ = 0.48 eV). The band gap is 1.24 eV for H-I and 0.91 eV for H-II (Fig. 4). Unlike BAs/MoSi_2_N_4_, which is type-I at equilibrium and reaches a type-II alignment only under vertical strain,^45^ H-II is intrinsically type-II. The contrast between H-I and H-II shows that the chalcogen termination facing BAs controls the alignment type, in line with the stacking-dependent type-I/type-II switching reported for AsP/MoSi_2_P_4_.^29,39,46^

**Fig. 4:**
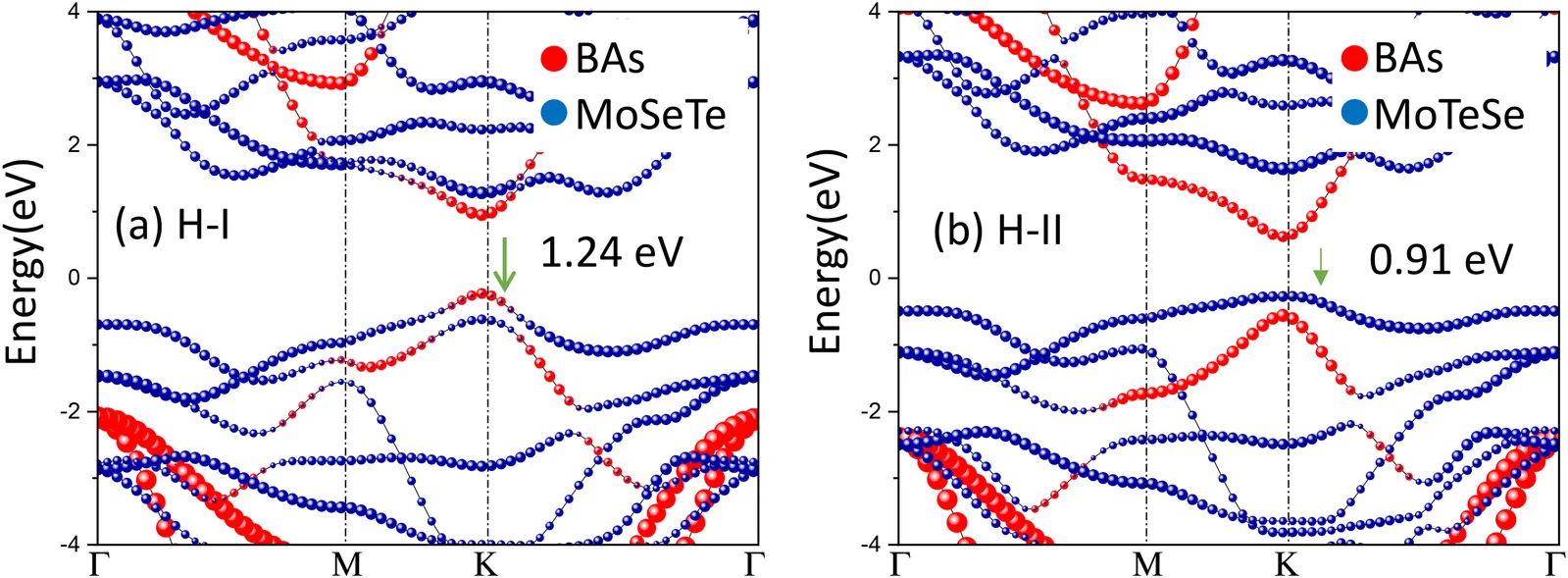
Layer-projected band structures of the (a) H-I and (b) H-II heterostructures at the HSE06 level. Blue (red) denotes states localised on the Janus MoSeTe/MoTeSe (BAs) layer.

When Se or Te vacancies were created in H-I or H-II, the interlayer band gap was further reduced and the band structure became indirect (Fig. S9). Specifically, for Se and Te vacancies in the H-I structure, the band gap values decreased from 1.24 to 0.95 eV and 1.10 eV, respectively. Similarly, when these vacancies were introduced in the H-II structure, the band gap values further decreased from 0.91 to 0.46 eV and 0.88 eV, respectively. Upon vacancy introduction, all heterostructures become indirect-gap semiconductors. The vacancy-induced reduction in interlayer band gap and the transition to indirect character have direct implications for carrier transport and photocatalytic charge separation, discussed below. Chalcogen vacancies are the most common native defects in transition-metal dichalcogenides (∼10^13^ cm^−2^ in as-grown monolayers),^47^ and their density can be tuned by Ar-plasma treatment,^48^ electron or ion irradiation,^49^ thermal annealing or electrochemical desulfurisation.^50^ Since Janus layers are themselves obtained by chalcogen substitution on one face of a parent monolayer,^13^ face-selective vacancy formation is in principle achievable. The concentration modelled here (≈6% of one chalcogen layer) is within the experimentally accessible range.

### Carrier mobilities

Within the framework of the effective mass approximation (EMA), deformation potential (DP) theory has been a widely used approach to investigate carrier mobility (*μ*) in 2D materials. The carrier mobility is defined as:^6,35^2
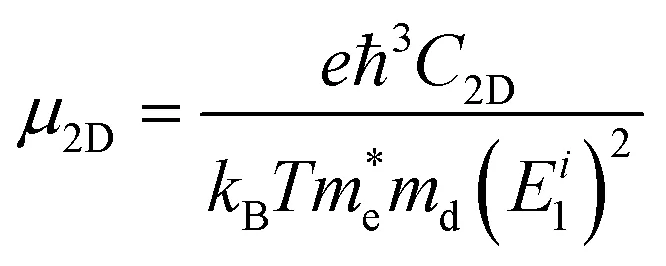
where *m*_d_ is the average effective mass, which is defined as 
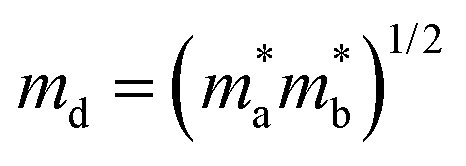
, 
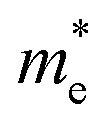
 is the carrier effective mass, *T* is the temperature, and *E*^*i*^_1_ is the DP constant. *E*^*i*^_1_ describes the shift of the top of the valence band for holes and the bottom of the conduction band for electrons induced by the strain in the range of ±1.5%. The value of *E*^*i*^_1_ is given by *E*^*i*^_1_ = Δ*E*_*i*_/(Δ*l*/*l*_0_) (see Fig. S2 in the SI for details). Moreover, in eqn (2), *C*_2D_ is the elastic modulus of *a* and *b*, uniformly deformed crystals for emulating the lattice distortion activated by the strain. For the 2D structure, the in-plane stiffness can be determined by *C*_2D_ = [∂^2^*E*/∂*δ*^2^]/*S*_0_, where *E* is the total energy of the supercell, *δ* is the applied uniaxial strain, and *S*_0_ is the area of the equilibrium supercell. The details for the calculation of *C*_2D_ are given in Fig. S1 and S2 of SI. Because the hexagonal lattices considered here are isotropic in-plane, *C*_2D_ and *E*_1_ are identical along the armchair and zigzag directions, and any directional dependence of *µ* arises solely from the effective masses. Where present, this asymmetry is expected to reduce the spatial overlap of photoinduced electrons and holes, contributing to suppressed recombination.

The BAs monolayer exhibits high carrier mobilities, with hole mobilities (16 565 and ≈19 880 cm^2^ V^−1^ s^−1^ along the armchair and zigzag directions) exceeding electron mobilities (6470 and ≈8010 cm^2^ V^−1^ s^−1^). This originates from the smaller hole deformation potential (*E*^h^_1_ = 2.02 eV *vs. E*^e^_1_ = 3.01 eV) and the light hole effective mass (0.20–0.24*m*_e_) of the As-4p-derived VBM (Table 2). These parameters agree with previous DP studies of BX monolayers, which reported |*E*^h^_1_| = 2.3 eV, |*E*^e^_1_| = 3.8 eV and *C*_2D_ = 121.5 N m^−1^ for BAs^51^ and mobilities exceeding 10^4^ cm^2^ V^−1^ s^−1^ for the BX family.^52^ By contrast, the MoSeTe monolayer shows an electron–hole asymmetry in the opposite sense: electron mobilities (1625 and ≈1820 cm^2^ V^−1^ s^−1^) are an order of magnitude larger than hole mobilities (156 and ≈180 cm^2^ V^−1^ s^−1^), reflecting the heavier hole effective mass 
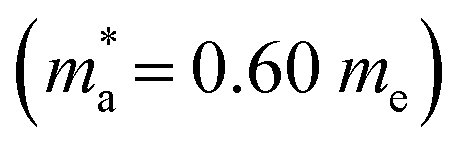
 and larger hole deformation potential (*E*^h^_1_ = 7.13 eV).^52^

**Table 2:** Carrier effective masses m* (in units of the free-electron mass *m*_e_) along the armchair (a) and zigzag (b) directions, deformation-potential constant *E*_1_ (eV), in-plane stiffness *C*_2D_ (N m^−1^) and carrier mobility *μ*^2D^ (cm^2^ V^−1^ s^−1^) at 300 K for electrons (e) and holes (h) of the BAs and Janus MoSeTe monolayers and the H-I and H-II heterostructures, obtained from deformation-potential theory (eqn (2)) at the PBE-D3 level. By the in-plane isotropy of the hexagonal lattices, *C*_2D_ and *E*_1_ are equal along *a* and *b*, and the directional dependence of *μ*^2D^ arises from the effective masses. Values include only longitudinal-acoustic-phonon scattering and therefore represent upper bounds to the intrinsic mobilities

System	Carrier	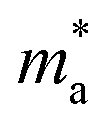 (*m*_e_)	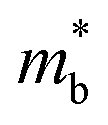 (*m*_e_)	*E*_1_ (eV)	*C*_2D_ (N m^−1^)	*μ* ^2D^_a_ (cm^2^ V^−1^ s^−1^)	*μ* ^2D^_b_ (cm^2^ V^−1^ s^−1^)
BAs	e	0.26	0.21	3.01	156.08	6470	8011
	h	0.24	0.20	2.02	156.08	16565	19878
MoSeTe	e	0.37	0.33	3.57	120.52	1625	1822
	h	0.60	0.52	7.13	120.52	156	180
H-I	e	0.31	0.25	5.16	279.52	2744	3402
	h	0.41	0.34	4.54	279.52	1977	2384
H-II	e	0.25	0.56	2.59	279.94	9990	4460
	h	0.21	0.39	4.68	279.94	4568	2460

Upon heterostructure formation, H-II exhibits the highest electron mobility of the pristine systems along the armchair direction (*µ*_a_ = 9990 cm^2^ V^−1^ s^−1^), driven by a low electron effective mass 
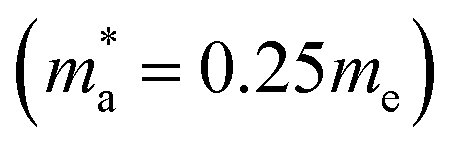
 and a small deformation potential (*E*^e^_1_ = 2.59 eV). H-I yields more moderate electron mobilities (2744 and ≈3400 cm^2^ V^−1^ s^−1^). The electron-to-hole mobility ratio (*µ*_e_/*µ*_h_, armchair), relevant to photoinduced carrier separation, is 2.2 for H-II and 1.4 for H-I, suggesting that mobility-driven suppression of recombination is more pronounced in H-II. These are DP estimates, which neglect optical-phonon and intervalley scattering and therefore represent upper bounds on the intrinsic mobilities.

Introducing chalcogen vacancies strongly increases the carrier effective mass along one in-plane direction while leaving the orthogonal direction comparatively unaffected. For electrons, 
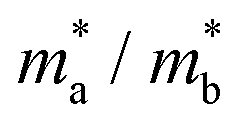
 = 0.53/5.77*m*_e_ in H-I with a Se vacancy and 0.17/3.78*m*_e_ in H-II with a Se vacancy. In H-I with a Te vacancy the anisotropy axis is reversed (1.49/0.32*m*_e_), and the H-II Te-vacancy system shows similar behaviour (0.17/2.62*m*_e_). These large directional masses are consistent with carrier localisation in the vacancy-induced flat bands (Fig. S9) and point to strongly anisotropic transport. Because these band edges are partly localised on the defect, the effective-mass approximation and DP theory are not reliable for them, and mobilities are therefore not reported for the vacancy-containing structures.

For comparison with related systems, Guan *et al.*^28^ reported DP electron mobilities of 134 and 3765 cm^2^ V^−1^ s^−1^ for BAs/WS_2_ and BAs/WSe_2_ heterostructures. The H-II electron mobility of ∼10^4^ cm^2^ V^−1^ s^−1^ (Table 2), obtained within the same framework, compares favourably with these values.^28^

### Optical properties

A prerequisite for photocatalytic activity is the ability to absorb photons within the visible spectrum (380–760 nm), which governs the upper bound of the photocurrent. The light absorption coefficient is as follows:3
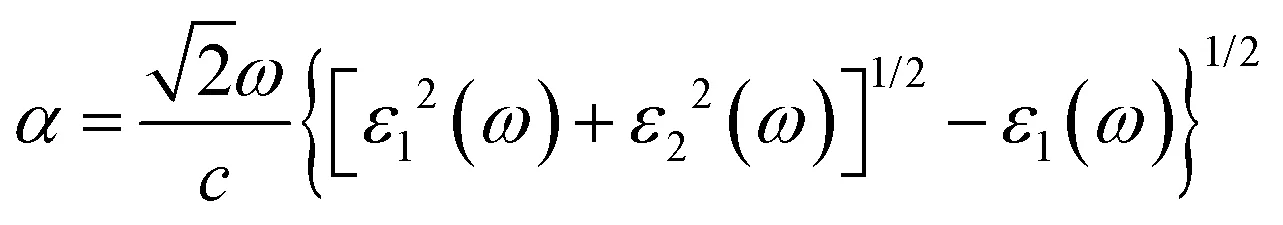
where *c* is the speed of light in vacuum, *ω* is the angular frequency, and *ε*_1_(*ω*) and *ε*_2_(*ω*) are the real and imaginary parts of the dielectric function, respectively.

Fig. 5 illustrates the light absorption coefficients with GW and HSE (Fig. S3) functionals for Janus-MoSeTe, BAs, H-I, and H-II heterostructures. In the visible spectrum, BAs exhibits the highest light absorption coefficients at wavelengths of 470 nm and 550 nm. In the ultraviolet spectrum, BAs shows significant absorption peaks at 150 nm, 210 nm, 280 nm, and 370 nm.^28^ The MoSeTe monolayer, on the other hand, has absorption peaks at 322 nm and 610 nm. Compared to BAs, the MoSeTe monolayer demonstrates a broader absorption range, effectively covering both the visible and ultraviolet regions. This wider absorption range makes MoSeTe particularly advantageous for applications requiring efficient light absorption across a broader spectrum.

**Fig. 5:**
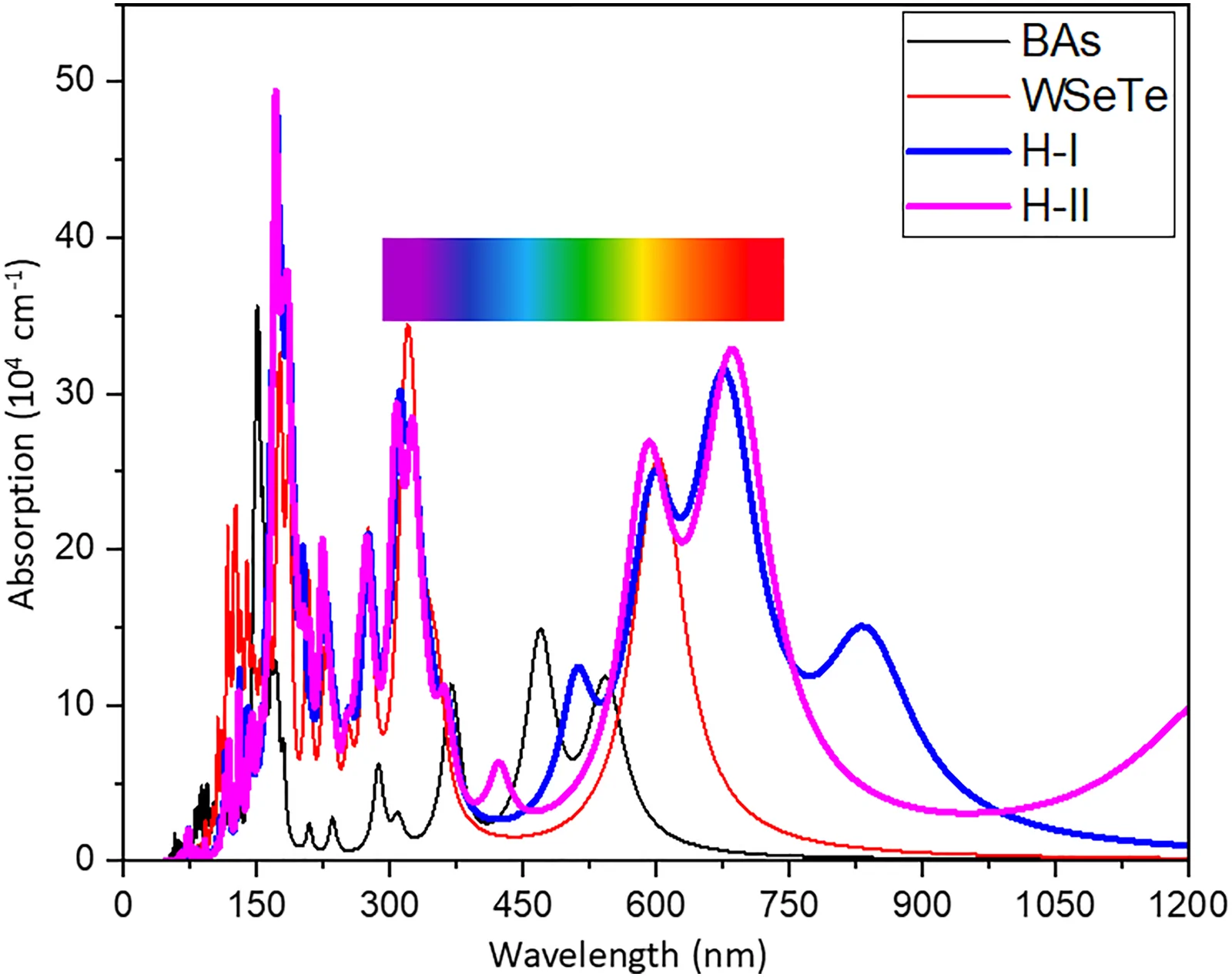
Optical absorption coefficients of the BAs and MoSeTe monolayers and the H-I and H-II heterostructures, calculated using the GW approximation.

The heterostructures introduce two additional peaks in the visible range compared to the monolayers: H-I exhibits a redshift at 630 nm, while H-II shows a blueshift at 600 nm with GW approximation. These heterostructures demonstrate superior light utilization in both the visible and ultraviolet regions, as evidenced by the significant increase in absorption intensity and the broadening of the light absorption spectrum. This enhanced absorption is attributed to several factors, including interlayer coupling, vacancies, and charge transfer. These elements collectively contribute to the increased light absorption coefficient, thereby improving the photocatalytic water splitting efficiency of the heterostructures.

### Photocatalytic and photovoltaic properties

Designing direct photocatalysts poses significant challenges, both experimentally and theoretically.^53^ DFT calculations can be employed to identify the type of heterostructure, such as type II configuration.^54^ Key factors to investigate include the built-in electric field (*E*_int_), charge density difference (Δ*ρ*), electron–hole recombination, effective masses, and interlayer band gaps. The Δ*ρ* at the interface was determined as follows:

4 Δ*ρ* = *ρ*_BAs/MoSeTe_ − *ρ*_BAs_ − *ρ*_MoSeTe_

where *ρ*_BAs/MoSeTe_, *ρ*_BAs_, and *ρ*_MoSeTe_ represent the charge density of the BAs/MoSeTe or BAs/MoTeSe vdWHs, BAs, and MoSeTe monolayers, respectively. The insets of Fig. 7 display the Δ*ρ* graphs. When two monolayers come into contact, electrons transfer across the interface due to the difference in Fermi levels. This charge movement continues until the Fermi levels reach equilibrium. According to eqn (4), the work function values can be determined from the known values of the Fermi levels.

5 *ϕ* = *E*_vac_ − *E*_F_

where *E*_vac_ and *E*_F_ are the vacuum and Fermi levels, respectively. The work function of MoSeTe is 5.19 eV, while that of BAs is 5.13 eV. The higher Fermi level of BAs (lower work function) drives electron transfer from the BAs monolayer to the Janus-MoSeTe or Janus-MoTeSe layer upon contact. Consequently, electrons accumulate on the surface of Janus-MoSeTe or Janus-MoTeSe, and holes gather on the BAs surface. As charge accumulates at the interface, a built-in electric field (*E*_int_) is generated from BAs to MoSeTe. This electric field will stop further charge transfer once the Fermi levels of both BAs and Janus-MoSeTe or Janus-MoTeSe monolayers reach equilibrium, resulting in the formation of heterostructures (H-I and H-II). Conversely, the polar systems Janus-MoSeTe and Janus-MoTeSe possess an intrinsic electric field that opposes and neutralizes the built-in electric field (*E*_int_), resulting in a net effective electric field (*E*_eff_) across the heterostructure. The electrostatic potential (*D*_ϕ_) determines the *E*_eff_ value, which is as follows:6
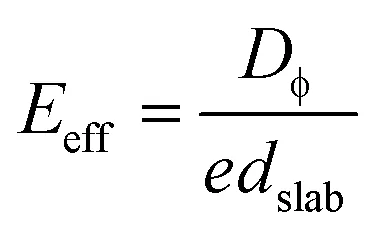
where *e* is the elementary charge and *d*_slab_ is the total slab thickness of the heterostructure (distinct from the interlayer spacing *d* reported in Table 1). The electrostatic potential difference (*D*_ϕ_), obtained from the planar-averaged electrostatic potential profiles (Fig. 6), is 0.43 eV for H-I and 0.75 eV for H-II.^28,38^ This *E*_eff_ produced in the heterostructure could effectively inhibit the recombination of photo-generated e^−^/h^+^ pairs and enhance carrier separation in H-I and H-II heterostructures, as shown in Fig. 7(g). The higher *E*_eff_ value of H-II compared to H-I indicates a slower electron–hole recombination rate in H-II, leading to superior catalytic performance and alignment with the type-II photocatalytic mechanism.^55,56^ H-II is therefore a type-II heterostructure, whereas H-I is type-I. In type-II heterostructures, efficient photocatalytic water splitting without mediators relies on staggered band alignment that facilitates directional charge transfer and spatial separation of electrons and holes across different layers. Optimal performance is achieved when interfacial recombination is suppressed and the band edge positions retain adequate driving force for both hydrogen evolution and oxygen evolution reactions. We note that Cao *et al.*^6^ reported a direct Z-scheme mechanism for the related MoTe_2_/BAs heterostructure. The distinct classification here arises from the different Janus monolayer composition and the resulting differences in interlayer charge transfer, as evidenced by the charge density difference (Δ*ρ*) and electrostatic potential profiles (Fig. 7).

**Fig. 6:**
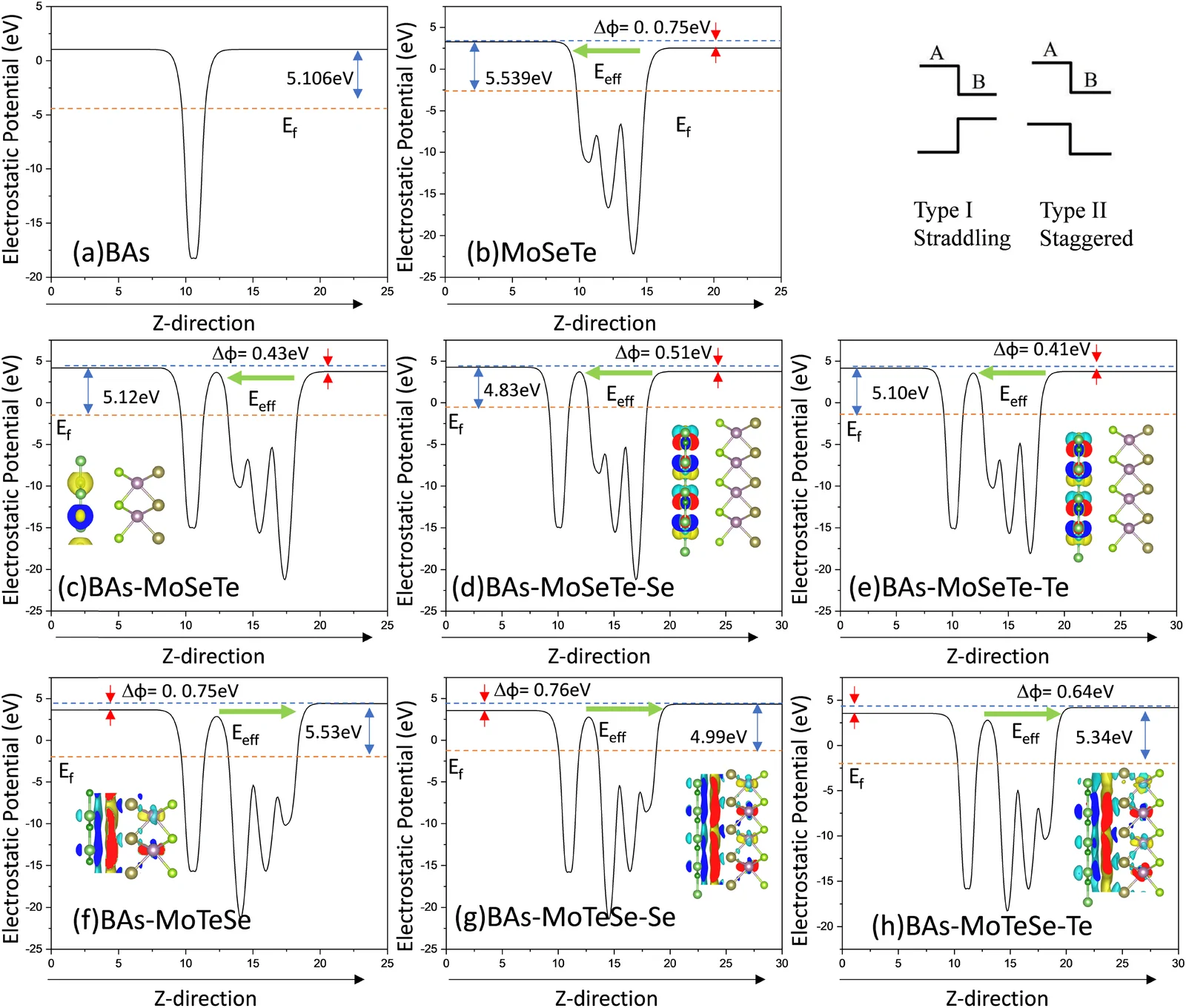
Planar-averaged electrostatic potential of (a) BAs, (b) MoSeTe, (c) H-I, (d) H-I with Se vacancy, (e) H-I with Te vacancy, (f) H-II, (g) H-II with Se vacancy, and (h) H-II with Te vacancy. Insets show the charge density difference (Δ*ρ*) isosurfaces; yellow (cyan) regions denote electron accumulation (depletion).

**Fig. 7:**
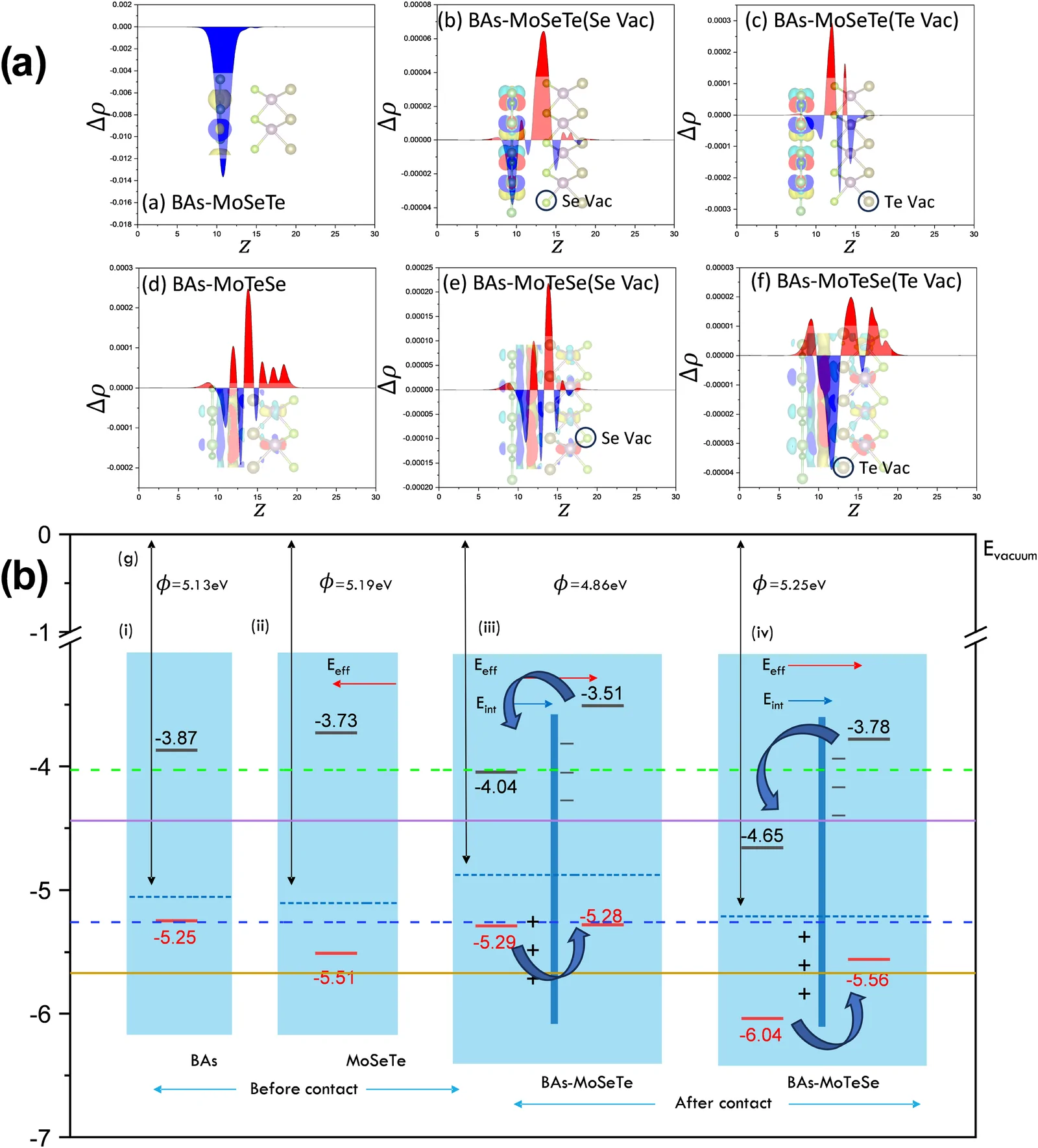
(a) Band-edge alignments of BAs, MoSeTe, H-I, and H-II heterostructures relative to the water redox potentials at pH 0 and pH 7. (b) Schematic representation of the photocatalytic charge-transfer mechanism for the pristine and vacancy-containing heterostructures.

For effective water decomposition catalysis, it is essential that the band edges of the photocatalyst align with the water redox potentials. This alignment ensures that the photocatalyst can efficiently drive the necessary redox reactions. Additionally, water splitting on the surface of heterostructures can only occur if the VBM of semiconductor-II is lower than the oxidation potential of O_2_/H_2_O, and its CBM is higher than the reduction potential of H^+^/H_2_. This specific alignment facilitates the separation and transfer of charge carriers, which is crucial for the water splitting process. To further understand this mechanism, we investigated the band alignments of the H-I and H-II heterostructures. The results of this investigation are presented in Fig. 7, which also includes the band alignments of BAs and MoTeSe monolayers for comparison. The reduction potential of H^+^/H_2_ and the oxidation potential of O_2_/H_2_O vary with pH levels. The specific values of these potentials at different pH levels are provided to give a comprehensive understanding of how the band alignments of the photocatalysts interact with the redox potentials under various conditions. This detailed analysis helps in optimizing the design and performance of photocatalysts for water decomposition applications.7
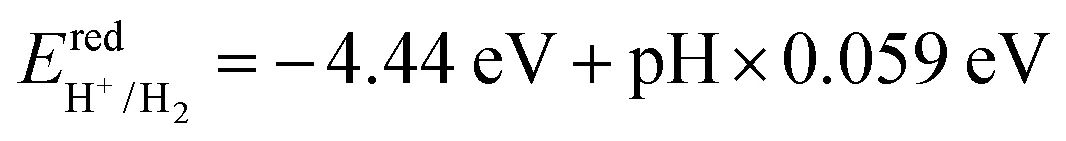
8
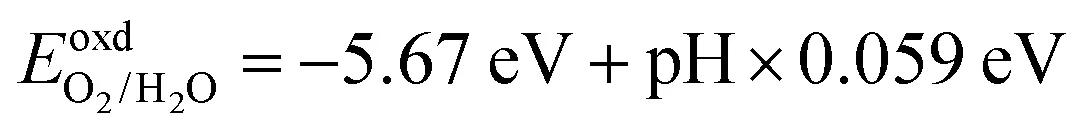
4.44 eV and −5.67 eV are reduction and oxidation potentials at a pH of zero, while at a pH of 7, the reduction and oxidation potentials are −4.03 eV and −5.26 eV, respectively. Although the isolated BAs and MoSeTe monolayers straddle the water redox potentials, in the heterostructures the type-II band edges do not: the CBM of H-II (−4.65 eV) lies below the H^+^/H_2_ level, and the VBM of H-I (−5.28 eV) lies above the O_2_/H_2_O level at pH 0 (Fig. 8). The mechanism of the photocatalytic process and the modification of photoexcited carriers for H-I and H-II heterostructures, as well as with Se and Te vacancies, are also depicted in Fig. 7. The heterostructure absorbs energy when it interacts with light, which causes electrons in the VBM to go to the CBM, creating holes in the VBM and electrons in the CBM. Upon photoexcitation, electrons and holes are generated in both layers. Due to the type-II band alignment, electrons migrate to the conduction band minimum (CBM) of one layer (*e.g.*, BAs), while holes accumulate at the valence band maximum (VBM) of the Janus MoSeTe or Janus MoTeSe layer, leading to effective spatial separation of charge carriers. The interlayer band gap governs the recombination behavior. In H-I, the interlayer band gap of 1.24 eV, along with a relatively small band offset (Δ*ϕ* = 0.43 eV), facilitates faster electron–hole recombination. After removing the Se atom in the H-I and H-II heterostructures, the band offsets (Δ*ϕ* = 0.51 eV and 0.76 eV) increase, which enhances carrier separation and suppresses recombination. However, the reduction in interlayer band gap (0.95 eV and 0.46 eV) may counteract this effect by providing energetically favorable recombination pathways. Therefore, the overall recombination dynamics result from a competition between band offset and interlayer gap. Similarly, introducing Te vacancies in the H-I and H-II heterostructures reduces the interlayer band gap (*e.g.*, from 1.24 eV to 1.10 eV and from 0.91 eV to 0.88 eV). In general, a reduction in interlayer band gap can facilitate electron–hole recombination, as it lowers the energy barrier for carrier recombination. However, the corresponding increase in band offset (Δ*ϕ* = 0.41 eV and 0.64 eV) is expected to promote charge carrier separation by providing a larger driving force for electron and hole transfer into different layers. Compared to Te vacancies, Se vacancies induce larger band offsets, leading to more efficient carrier separation and reduced recombination. Therefore, Se vacancies are more effective than Te vacancies for promoting spatial separation of photoexcited carriers. The introduction of Se or Te vacancies generates localised electronic states near the VBM and CBM (Fig. S9), which may act as trapping centres that spatially localise charge carriers and reduce their interlayer mobility. In principle, such localisation could suppress direct electron–hole recombination across the interface by inhibiting carrier transport between layers. However, localised defect states can also mediate non-radiative Shockley–Read–Hall recombination, in which electrons and holes are captured sequentially at the trap site, potentially accelerating rather than suppressing recombination. The net effect depends on the trap-state energy, capture cross-section, and carrier concentration—quantities that require carrier dynamics calculations beyond the scope of the present study. The band structure and effective mass data presented here are consistent with reduced interlayer carrier mobility upon vacancy introduction, but whether this translates into enhanced photocatalytic efficiency remains an open question that warrants further investigation using methods such as non-adiabatic molecular dynamics. The larger band offsets, potential drop and electron-to-hole mobility ratio of H-II relative to H-I consistently indicate more effective carrier separation in H-II; quantifying the corresponding transfer and recombination time scales is left for future work.

**Fig. 8:**
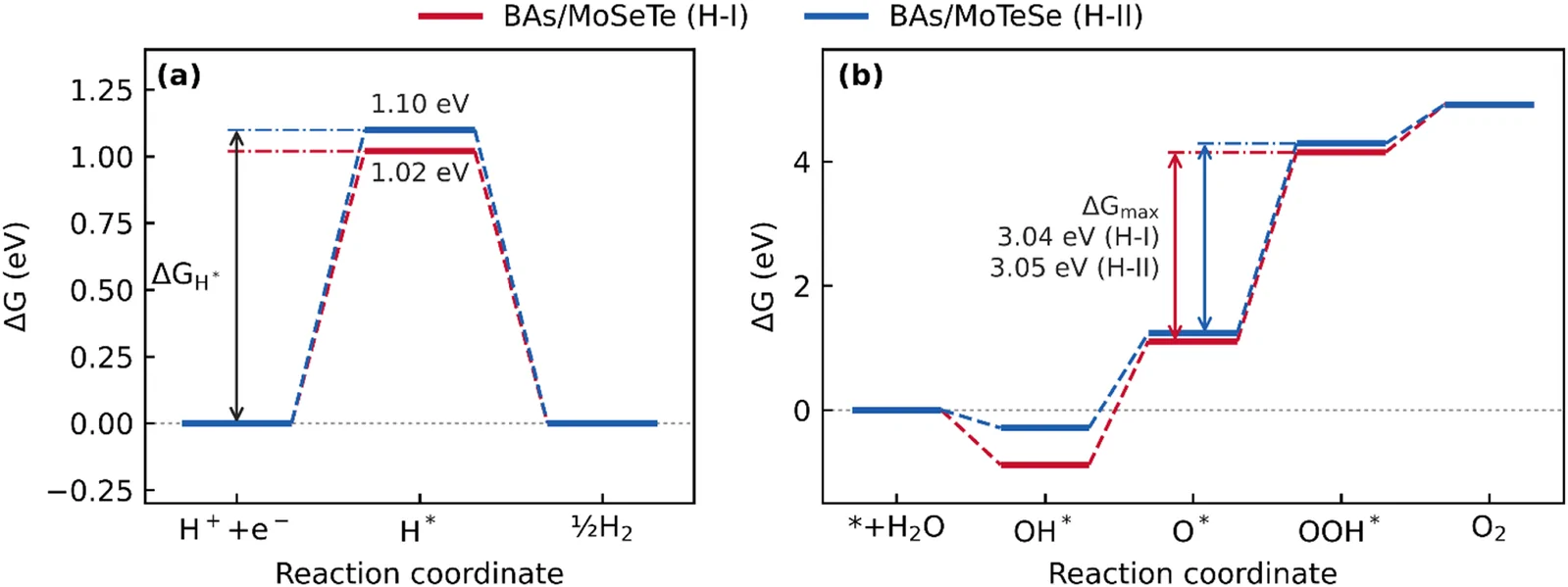
Gibbs free-energy diagrams for (a) the hydrogen evolution reaction and (b) the oxygen evolution reaction on the BAs/MoSeTe (H-I, red) and BAs/MoTeSe (H-II, blue) heterostructures at *U* = 0 and pH = 0. Arrows indicate the hydrogen adsorption free energy, Δ*G*_H*_, and the potential-determining step of the OER, Δ*G*_max_.

The hydrogen evolution reaction (HER) and oxygen evolution reaction (OER) are critical processes in water splitting for hydrogen production. The Gibbs free energy of hydrogen adsorption (Δ*G*_H*_) is 1.1 eV for BAs-MoSeTe and 1.02 eV for BAs-MoTeSe (Fig. 8a), with BAs-MoTeSe marginally closer to thermoneutral. However, both values substantially exceed the |Δ*G*_H*_| < 0.2 eV range characteristic of efficient HER catalysts, indicating that neither pristine heterostructure is a competitive hydrogen evolution electrocatalyst under the conditions modelled. The OER free energy profiles (Fig. 8b) show similarly large overpotentials for both systems (Δ*G*_max_ ≈ 3.0 eV for the *O → *OOH step). These results suggest that while the band-edge positions of H-I and H-II satisfy the thermodynamic requirements for water splitting (Fig. 6), the surface reaction kinetics, as approximated by Δ*G*_H*_ and OER overpotentials, remain a limiting factor. Further surface engineering, such as edge-site exposure or co-catalyst loading, may be required to bring the catalytic performance into a practical range.

The most stable heterostructure configurations exhibit type-II band alignment, which is essential for efficient photovoltaic energy conversion. Their photovoltaic conversion efficiency (PCE) was estimated using the open-circuit voltage (*V*_OC_), short-circuit current (*I*_SC_), and fill factor (FF), which are key parameters for characterizing the performance of a photovoltaic device. The power conversion efficiency of the considered heterostructures was evaluated using eqn (9).9
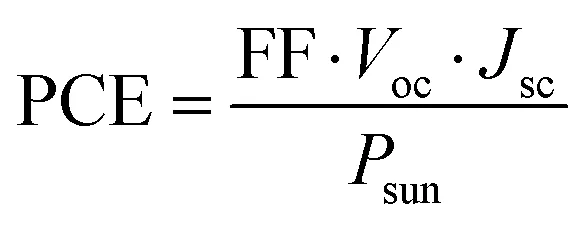
10
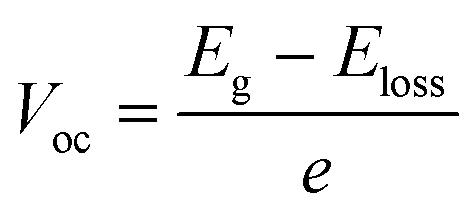
11
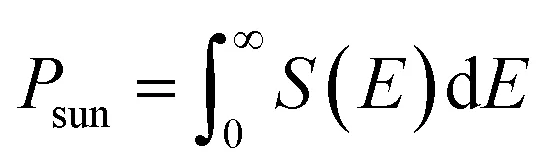
12
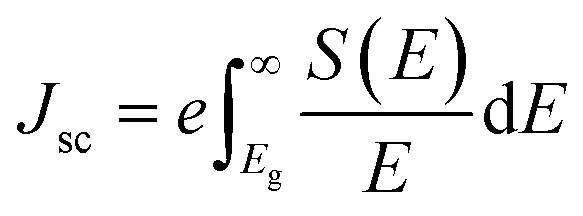
where *E*_g_ is the band gap of the heterostructure (1.24 eV for H-I and 0.91 eV for H-II), *E*_loss_ accounts for energy dissipation processes, *S*(*E*) is the solar power per unit area and photon energy (NREL AM1.5G spectrum), *V*_OC_ is the open-circuit voltage, *J*_SC_ is the short-circuit current density, *E* is the photon energy and FF is the fill factor. Adopting FF = 0.57 and *E*_loss_ = 0.2–0.3 eV, these assumptions yield PCE values of 20.4–22.5% (H-I) and 18.1–21.1% (H-II), with ideal short-circuit current densities of 38.0 and 52.1 mA cm^−2^, respectively. For comparison, a PCE of 17.03% was reported for the AsP/MoSi_2_P_4_ heterostructure under −4% compressive strain.^46^ Varying *E*_g_ by ±0.1 eV, a typical uncertainty of HSE06 band gaps, changes the PCE of H-I by less than 1 percentage point, owing to its proximity to the single-junction optimum, and that of H-II by up to ≈3 percentage points. These values assume an external quantum efficiency of unity above the band gap and neglect interfacial recombination, contact resistance and exciton binding; they should therefore be regarded as upper bounds. Device-level properties such as wavelength-resolved EQE and photoresponsivity require transport simulations beyond the scope of this work.

## Conclusions

This study reports a DFT investigation of the electronic, optical, and photocatalytic properties of BAs/Janus-MoSeTe (H-I) and BAs/Janus-MoTeSe (H-II) heterostructures. The stability of the heterostructures was confirmed through phonon spectrum analysis. Both pristine heterostructures adopt a type-II band alignment with direct band gaps of 1.24 eV (H-I) and 0.91 eV (H-II). Introducing Se or Te vacancies reduces the interlayer band gap and converts the structures to indirect semiconductors, with Se vacancies producing larger band offsets and more effective carrier separation than Te vacancies. Charge redistribution at the interface generates a built-in electric field that, under visible-light illumination, drives spatial separation of photoexcited electrons (accumulating in the CBM of the BAs layer) and holes (accumulating in the VBM of the Janus-MoSeTe or Janus-MoTeSe layer).

The calculated power conversion efficiencies of 25.25% (H-I) and 24.78% (H-II), estimated under the Shockley–Queisser framework with a fill factor of 0.57, are competitive with the highest reported theoretical values for 2D van der Waals heterostructures, confirming the strong photovoltaic potential of these systems. The band-edge positions of the heterostructures do not satisfy the thermodynamic criteria for overall water splitting within the type-II pathway, and the hydrogen adsorption free energies (Δ*G*_H*_ ≈ 1.0–1.1 eV) and OER overpotentials remain substantially above the thresholds required for efficient electrocatalysis. Realising the photocatalytic potential of these heterostructures will therefore require further surface modification, such as edge-site engineering or co-catalyst integration, to bring the reaction kinetics into a practical range.

Finally, since both BAs and Janus MoSeTe are non-magnetic, the K and K′ valleys of the present heterostructures remain degenerate; coupling the Janus layer to a magnetic substrate, as demonstrated for CrI_3_/MSi_2_P_4_,^57^ could provide an additional route to valley-contrasting functionality and is a direction for future work.

## Author contributions

Conceptualization of work: AGN and DDT; computation: HB, AGN; data analyses: AGN; data dissemination and graphics: HB, AGN; writing of the manuscript: HB, AR, AGN, and DDT; project support: DDT.

## Conflicts of interest

There are no conflicts to declare.

## Supplementary Material

CP-OLF-D6CP03340G-s001

## Data Availability

The data supporting this article have been included as part of the supplementary information (SI). Supplementary information: structural parameters, phonon dispersions, *ab initio* molecular dynamics energy profiles, the strain–energy and band-edge–strain data underlying the deformation-potential and in-plane stiffness fits (Fig. S1 and S2), and the HSE06 absorption spectra (Fig. S3). All computational parameters required to reproduce the reported results are given in the Computational details section of the article. See DOI: https://doi.org/10.1039/d6cp03340g.
