# Supplementary material for: Two-dimensional van der Waals heterostructures of BAs/MoSeTe and BAs/MoTeSe for photocatalytic and photovoltaic applications
Source: Phys Chem Chem Phys. 2026 Oct 21. Online ahead of print. doi: 10.1039/d6cp03340g (PMC13742165; doi:10.1039/d6cp03340g)
Supplement: Supplementary file 1 [file CP-OLF-D6CP03340G-s001.pdf]

Supplementary Information (SI) for Physical Chemistry Chemical Physics.
This journal is © the Owner Societies

Two-Dimensional van der Waals Heterostructures of BAs/MoSeTe and BAs/MoTeSe for Photocatalytic and Photovoltaic Applications

Hanifan BiBi[1], Azeem Ghulam Nabi[2,5,*], Aman-ur-Rehman[3,4], and Devis Di Tommaso[5,*]

AFFILIATIONS

[1]Department of Physics, Allama Iqbal Open University, H-8, Islamabad, 44000, Pakistan

[2] Department of Physics, Hafiz Hayat Campus, University of Gujrat, Gujrat, 50700, Pakistan

[3] Department of Nuclear Engineering, Pakistan Institute of Engineering & Applied Sciences, Nilore, Islamabad 45650, Pakistan

[4]Center for Mathematical Sciences, Pakistan Institute of Engineering & Applied Sciences, Nilore, Islamabad 45650, Pakistan

[5] School of Physical and Chemical Sciences, Mile End Road, London, Queen Mary University of London, E1 4NS, U.K.

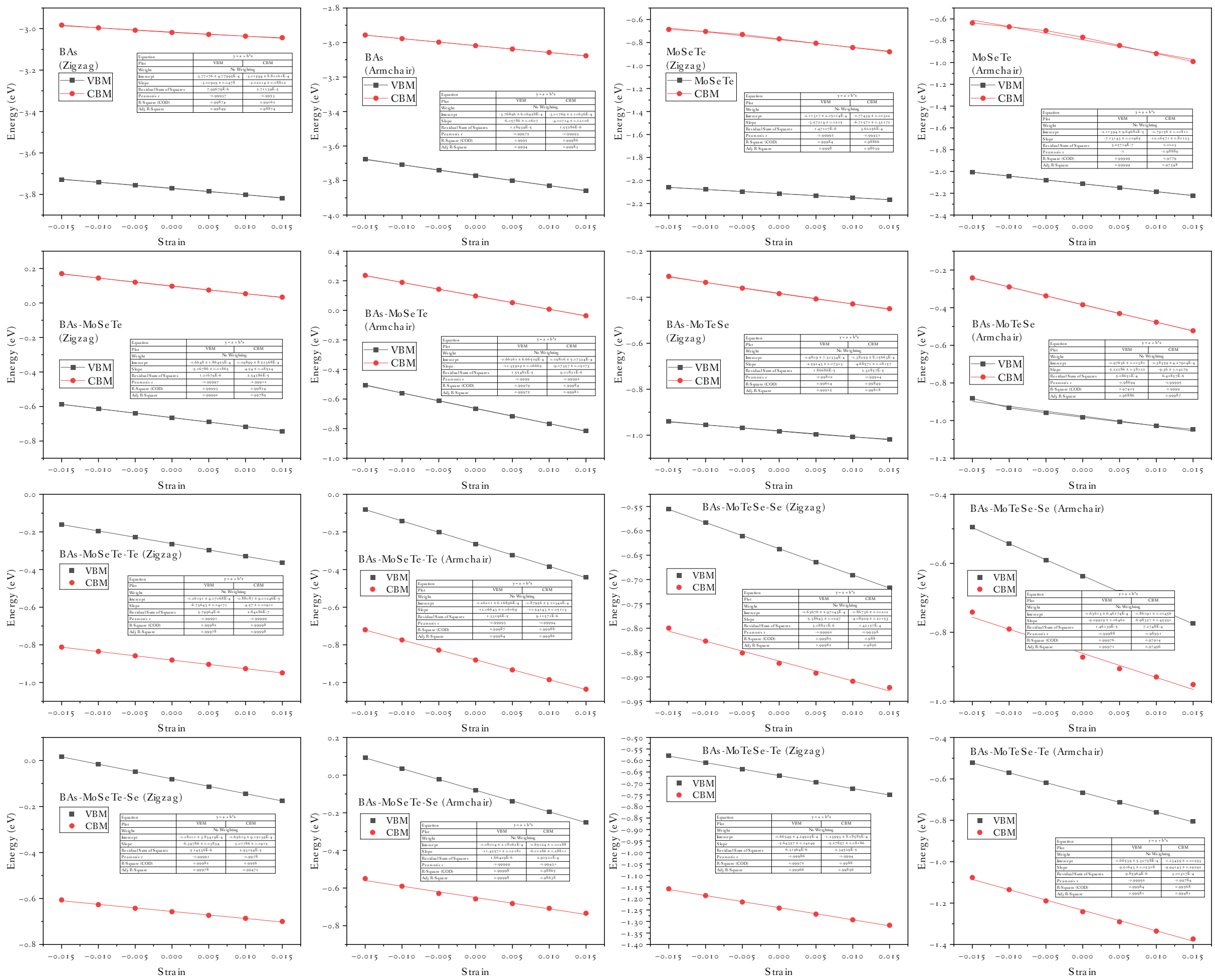

Figure S1. Band-edge energy shifts (CBM and VBM) under uniaxial strain with linear fittings used to extract deformation potential constants and calculate electron and hole mobilities.

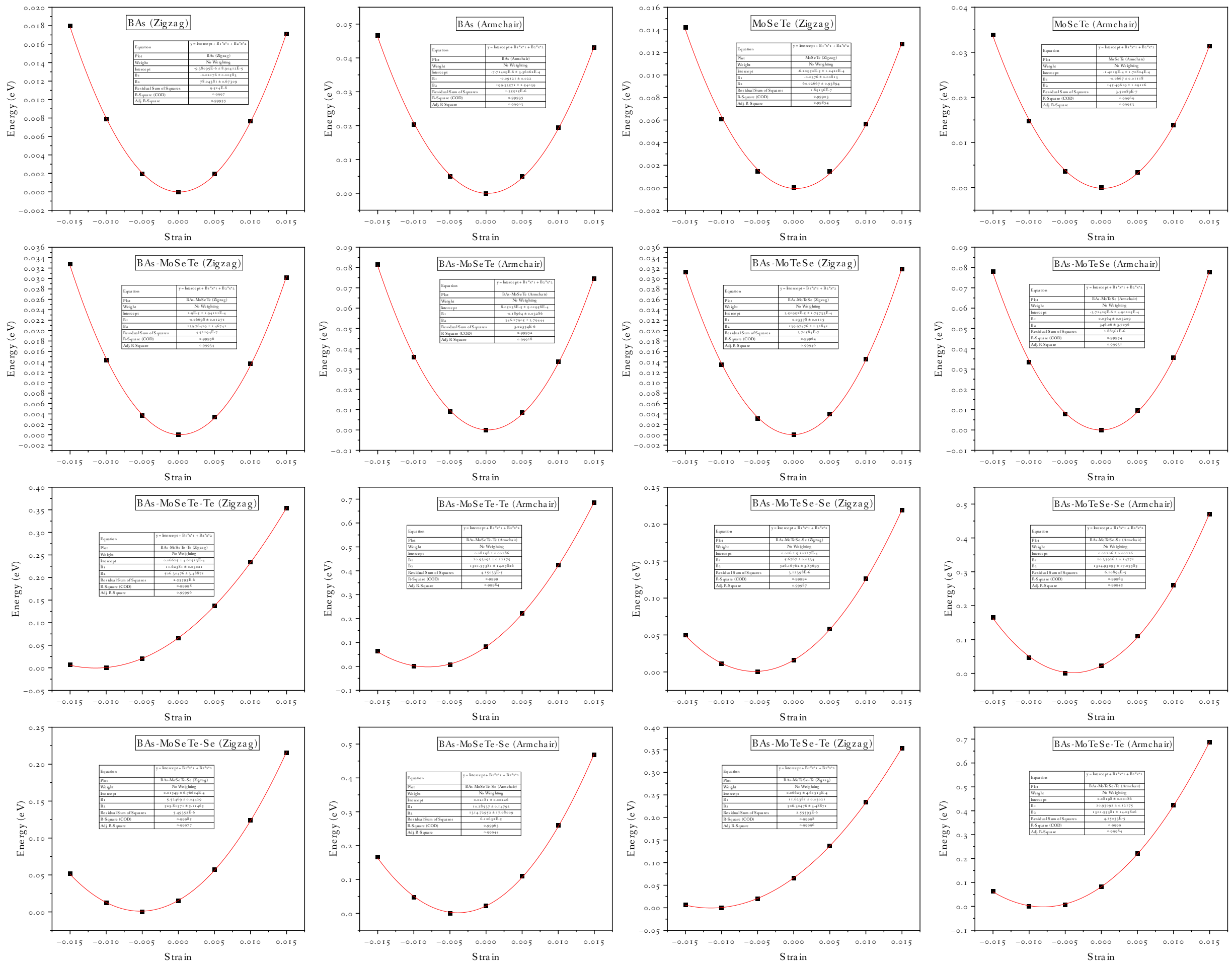

Figure S2. Parabolic fitting of total energy as a function of applied strain along the armchair and zigzag directions of the BAs/MoSeTe heterostructure. The curvature of the fitted curves yields the in-plane elastic modulus (C_{2D}), providing a measure of the mechanical stiffness and a key input parameter for carrier mobility calculations.

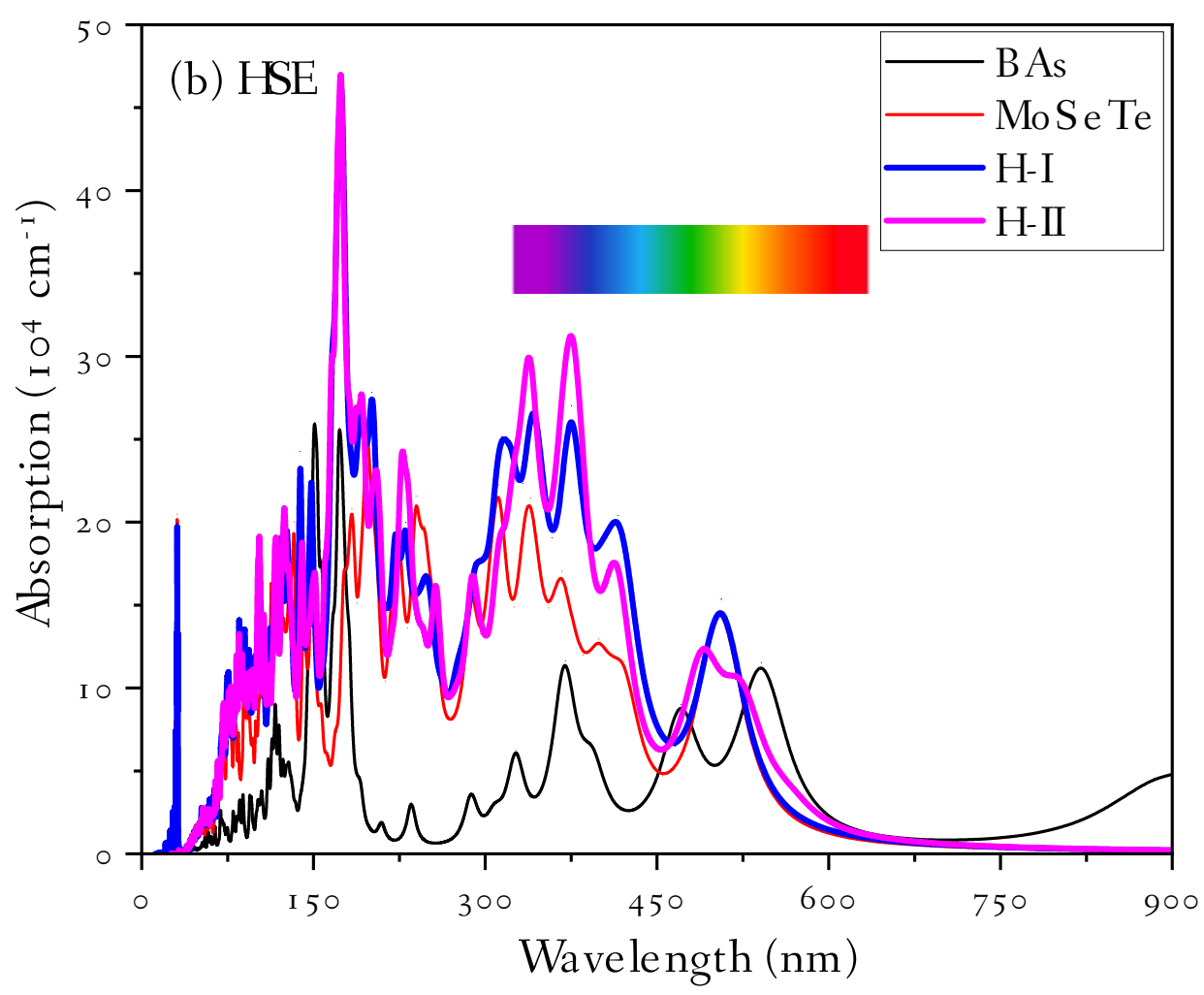

Figure S3. The absorption coefficient of the monolayers BAs, MoSeTe, and two different heterostructures.

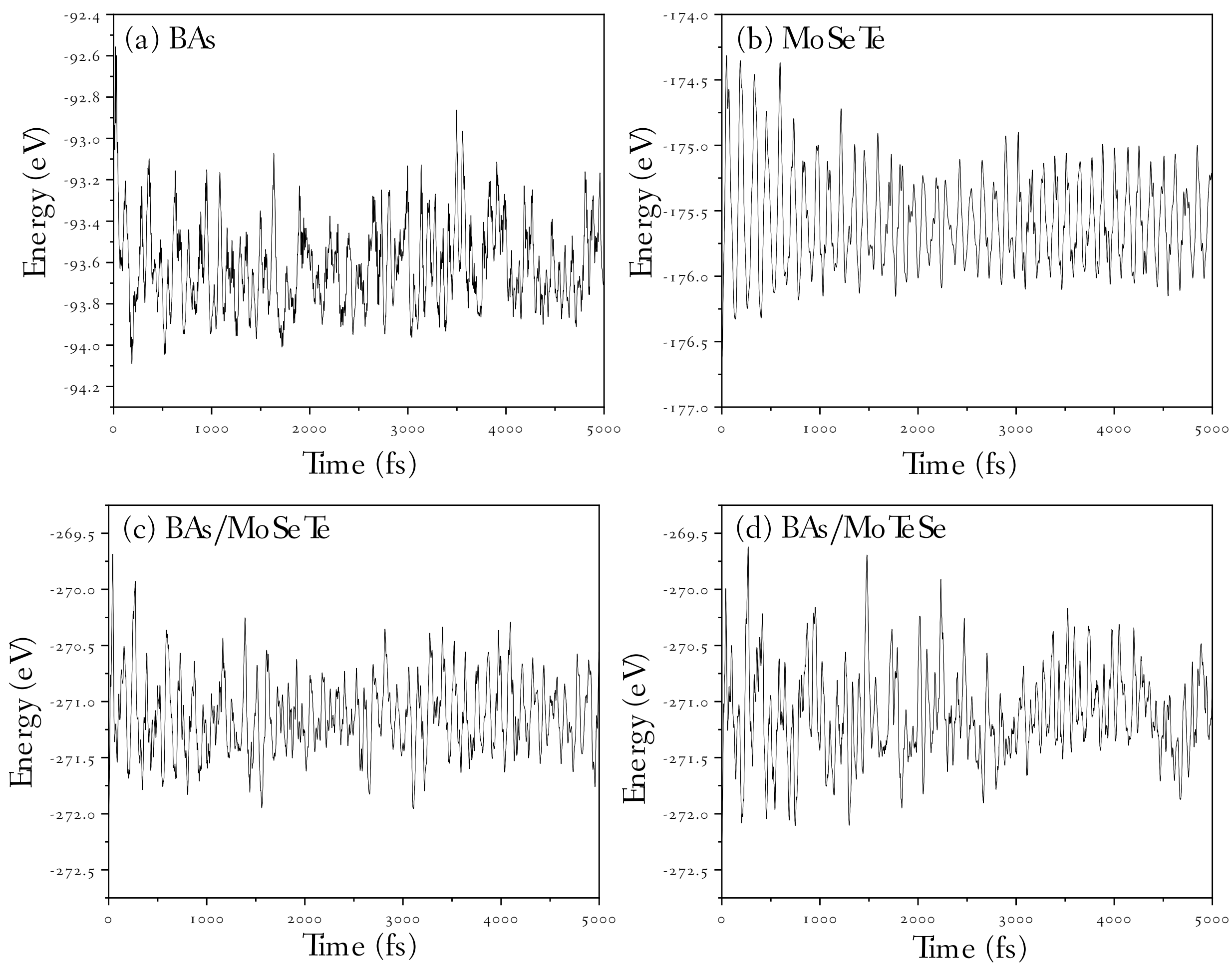

Figure S4. Plot of the total energy during the AIMD simulations of **(a)** BAs, **(b)** MoSeTe, **(c)** BAs-MoSete, and **(d)** BAs-MoTeSe calculated with a 4×4×1 supercell.

AIMD simulations configurations

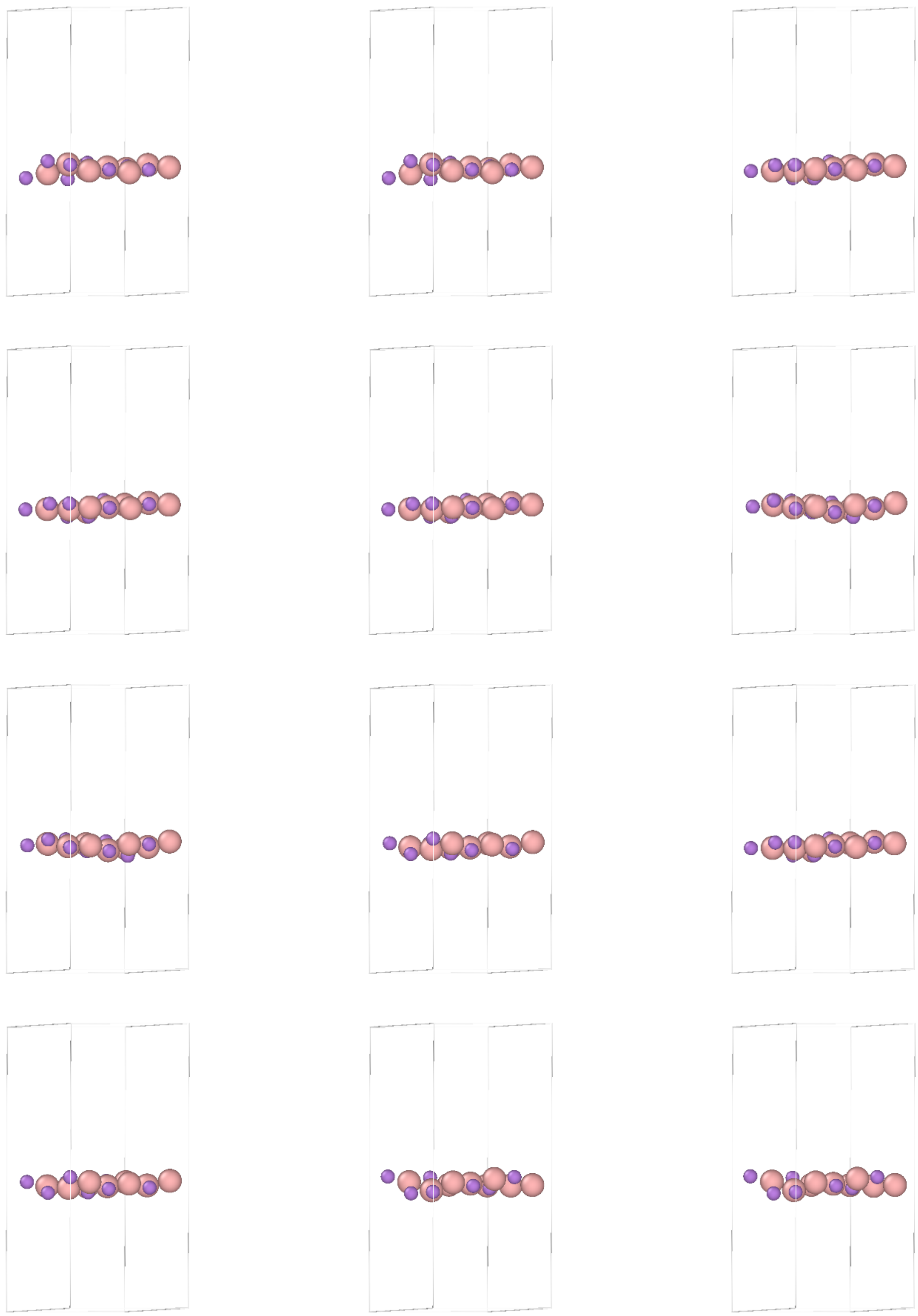

Figure S5(a): AIMD configurations for BAs monolayer

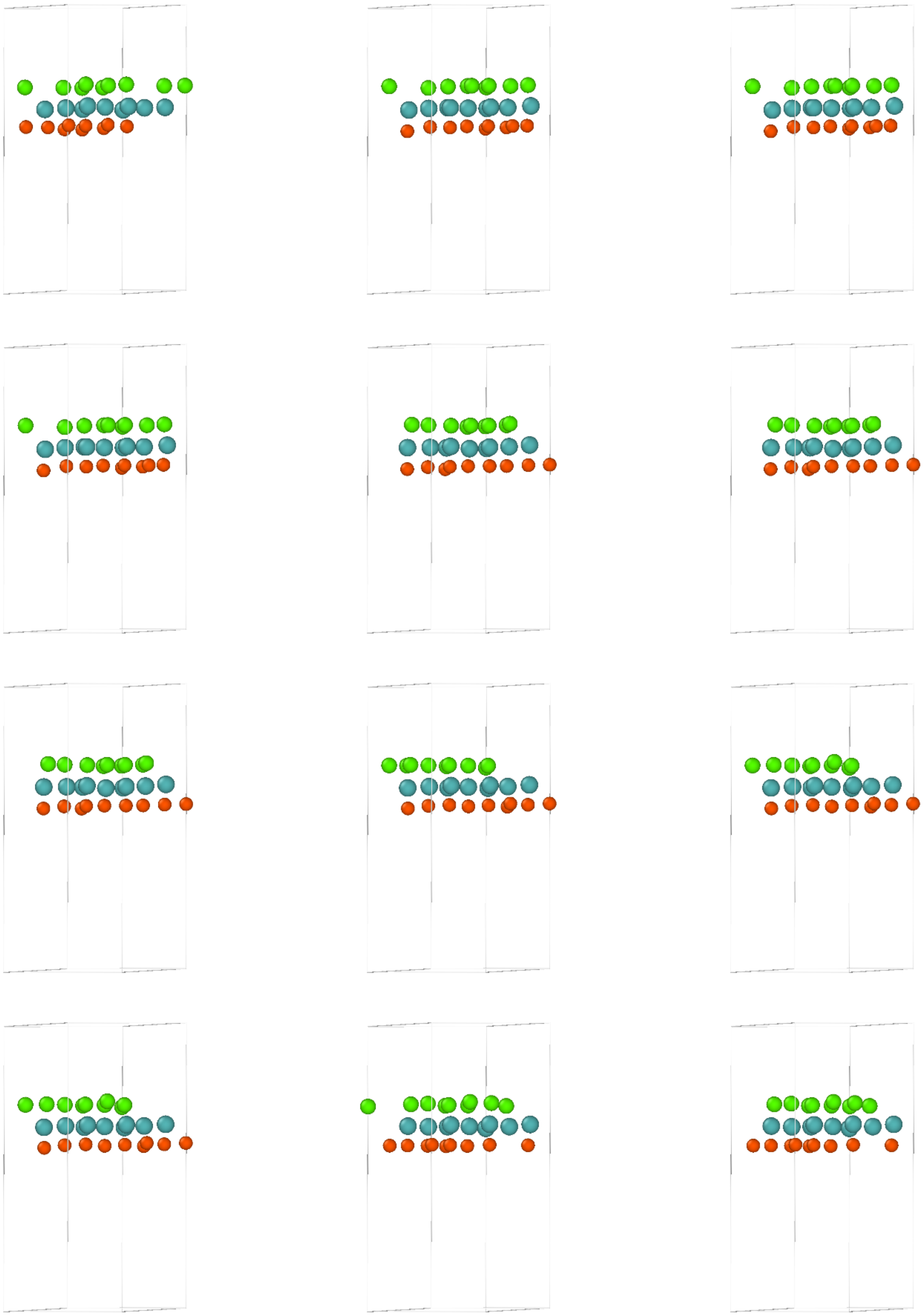

Figure S6: AIMD configurations for MoSeTe monolayer

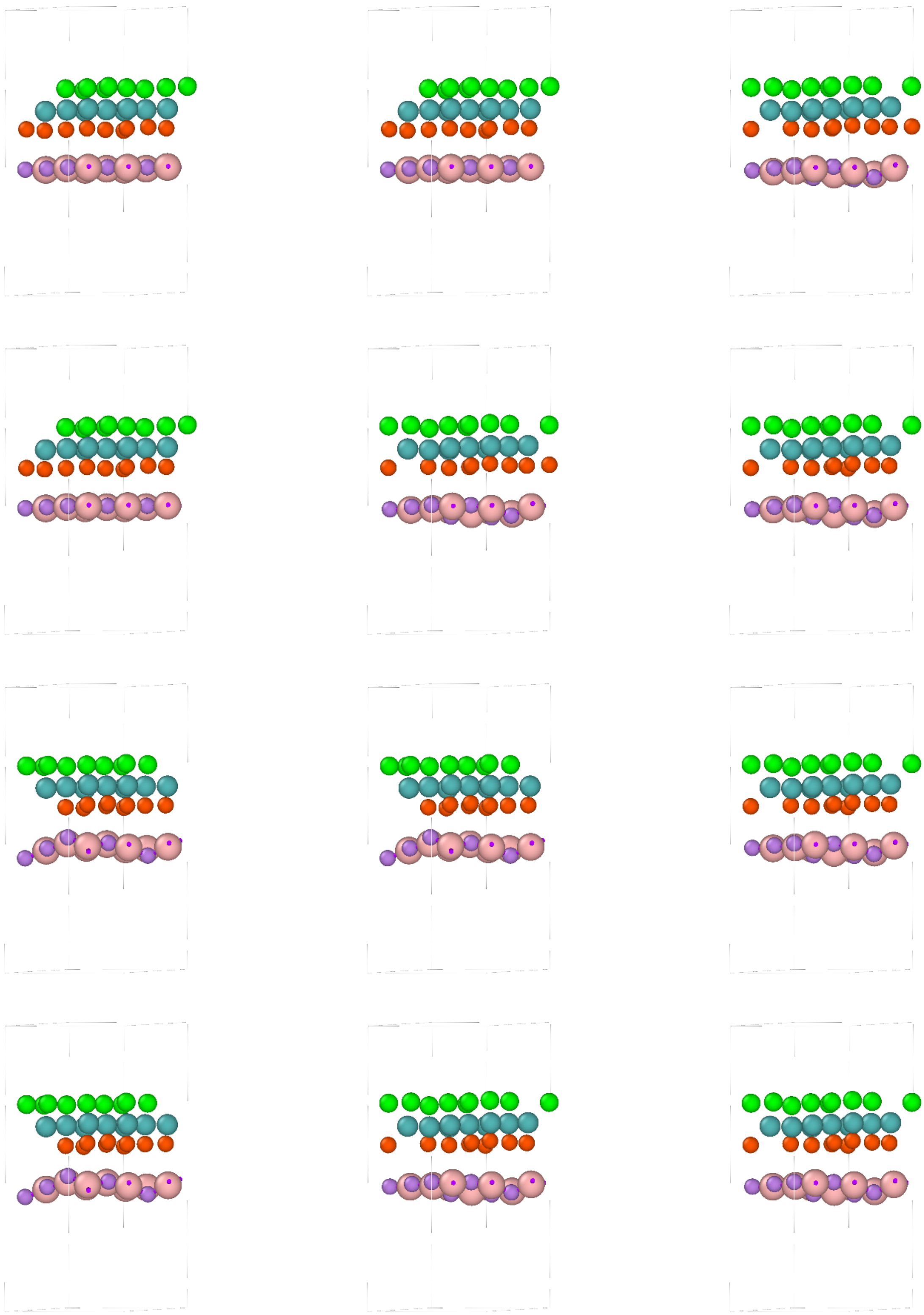

Figure S7: AIMD configurations for BAs/MoSeTe monolayer

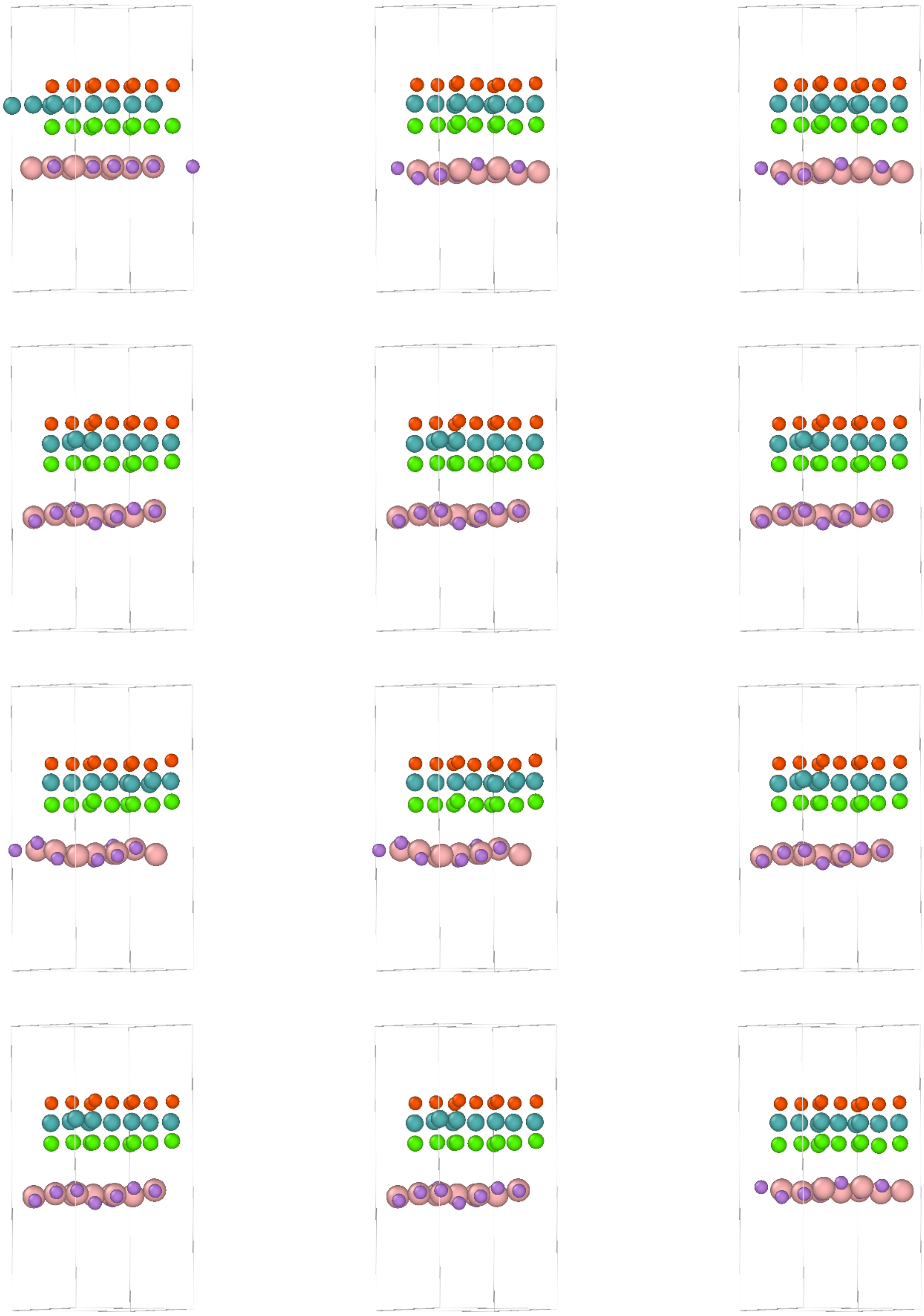

Figure S8: AIMD configurations for BAs/MoTeSe monolayer

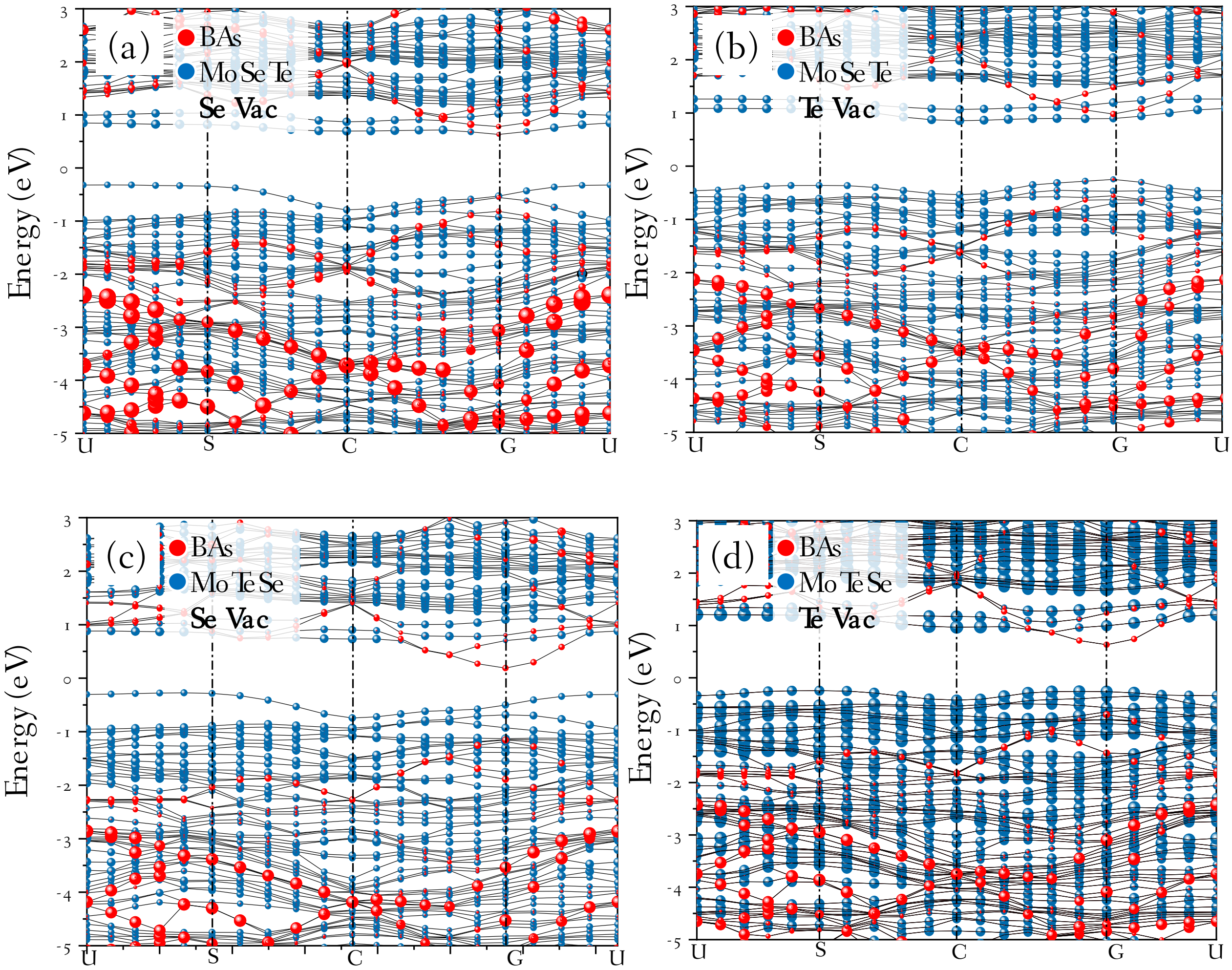

Figure S9. Layer-projected band structures of the vacancy-containing heterostructures at the HSE06 level: **(a)** H-I with Se vacancy, **(b)** H-I with Te vacancy, **(c)** H-II with Se vacancy, and **(d)** H-II with Te vacancy.
